# Supplementary figures and images for: Rare protein-altering variants in ANGPTL7 lower intraocular pressure and protect against glaucoma
Source: PLoS Genet. 2020 May 5;16(5):e1008682. doi: 10.1371/journal.pgen.1008682 (PMC7199928; doi:10.1371/journal.pgen.1008682)

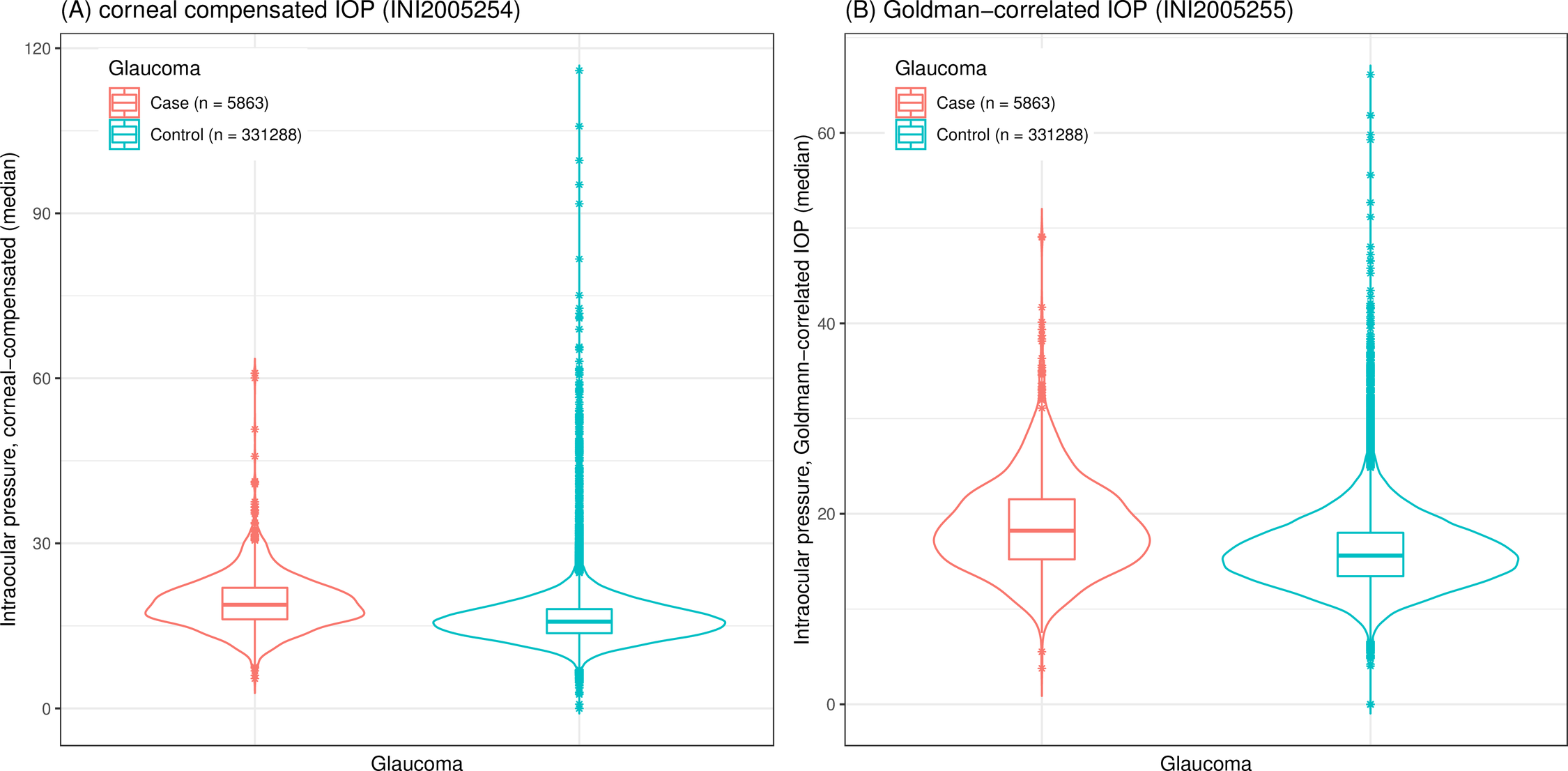

Supplement: S1 Fig — Phenotype distributions of the corneal-compensated (A) and Goldman-correlated (B) IOP measurements (the median of left and right eyes) stratified by glaucoma disease status in unrelated White British in UK Biobank displayed as a Tukey’s box plot overlapping on a violin plot. In the box plot, the middle bold horizontal line represents the median, the lower and upper hinges show the first and third quartiles, the lower and upper whiskers represent 1.5 * interquartile range from the hinges. The data points beyond whiskers are plotted individually. (TIF) [file pgen.1008682.s002.tif]

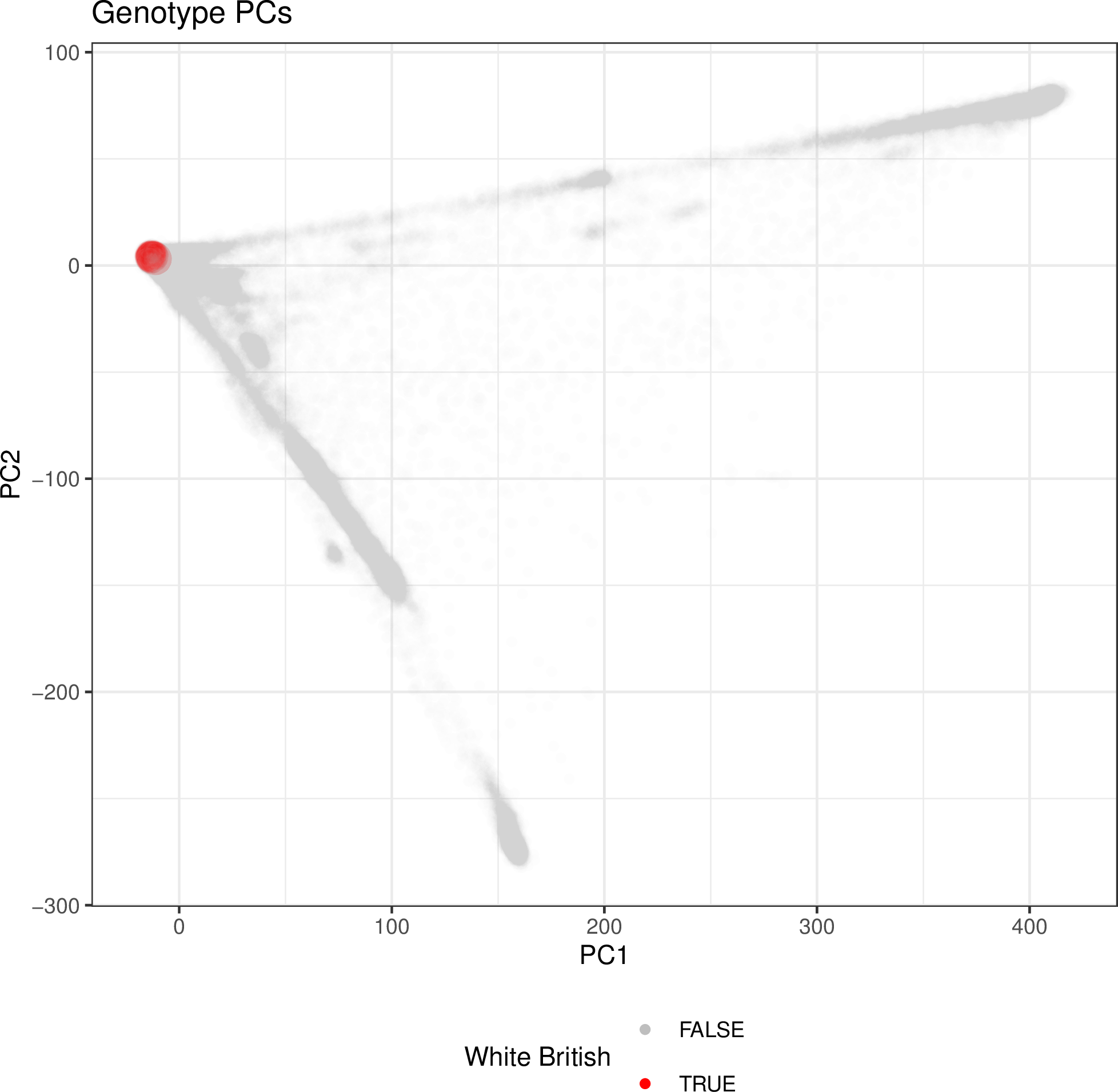

Supplement: S2 Fig — The identification of unrelated White British individuals in UK Biobank. The first two genotype principal components (PCs) are shown on the x- and y-axis and the identified unrelated White British individuals (Methods) are shown in red. (TIF) [file pgen.1008682.s003.tif]

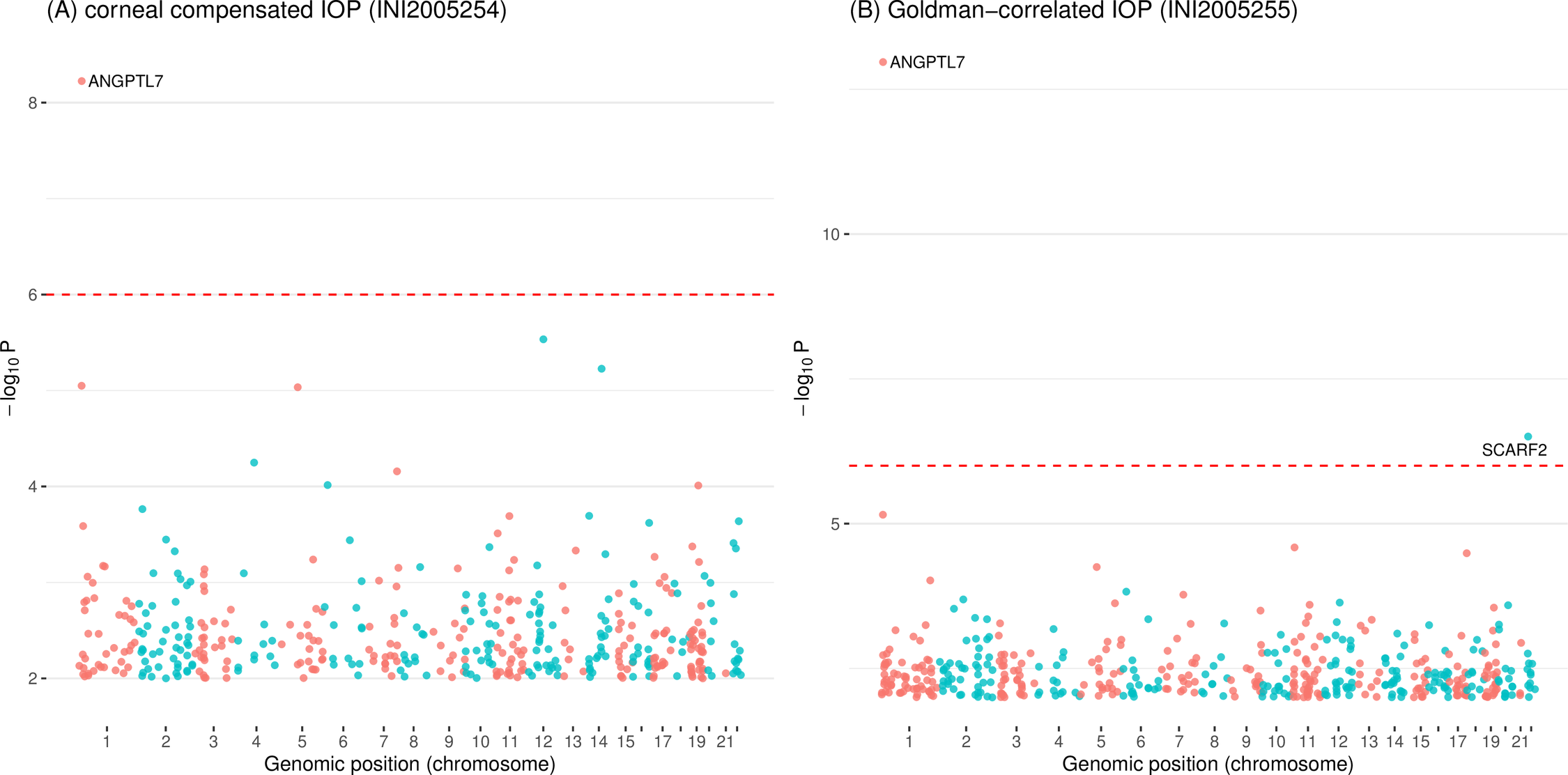

Supplement: S3 Fig — Genome-wide protein-altering variant association analysis of corneal compensated (A) and Goldman-correlated (B) intraocular pressure in UK Biobank. The rare (0.01% < MAF < 1%) protein-altering variants with P < 0.01 are shown. The red dashed horizontal line represents the genome-wide significance threshold (P = 10−6). The variants are shown in red (odd autosomes) or blue (even autosomes). The genomic coordinates of the variants are shown on the x-axis and the statistical significance of univariate analysis is shown on the y-axis. (TIF) [file pgen.1008682.s004.tif]

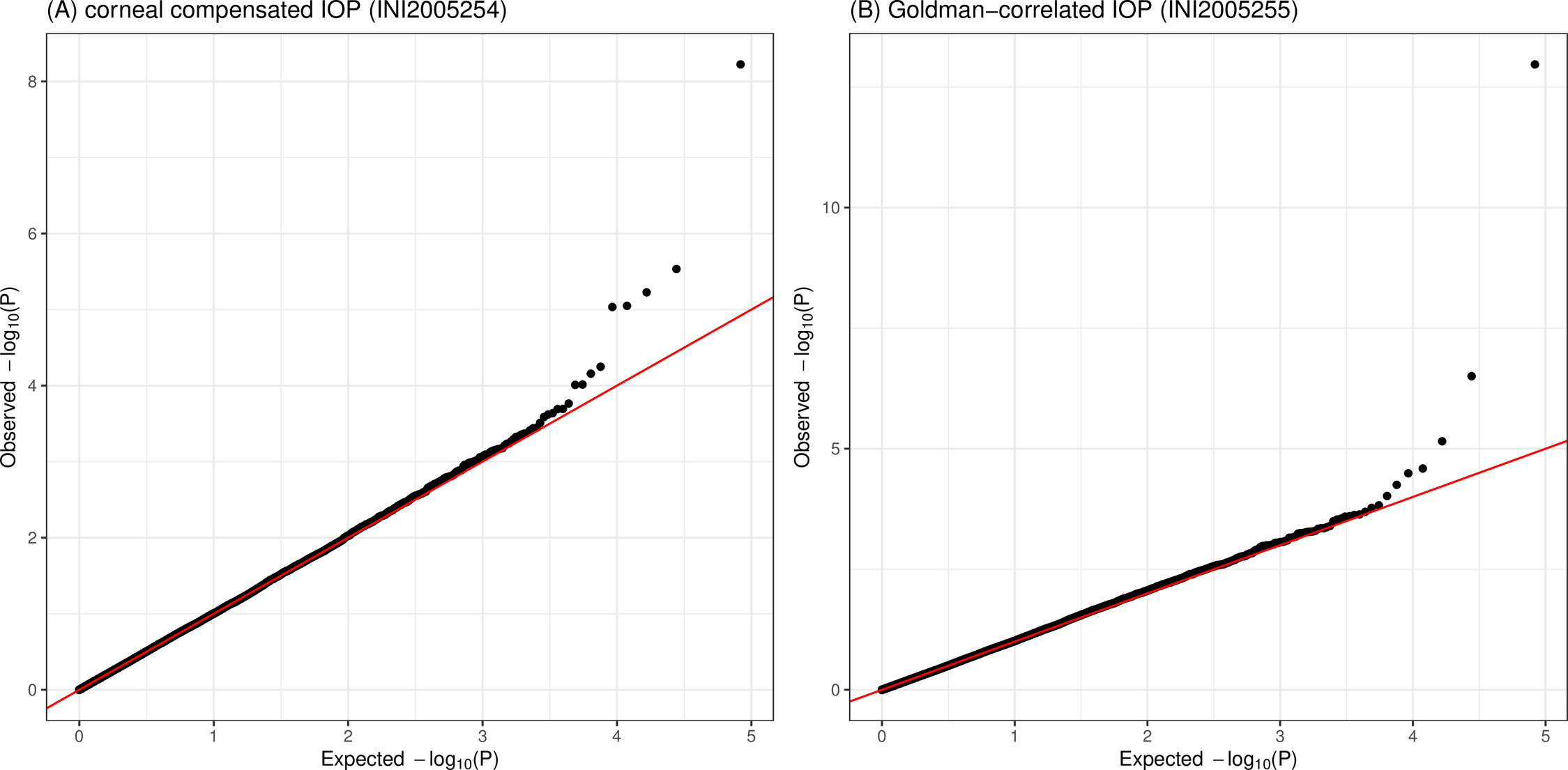

Supplement: S4 Fig — The protein-altering variant GWAS QQ plot for corneal compensated (A) and Goldman-correlated (B) intraocular pressure. The variants outside of MHC region with 0.01% < MAF < 1% are included in the analysis. (TIF) [file pgen.1008682.s005.tif]

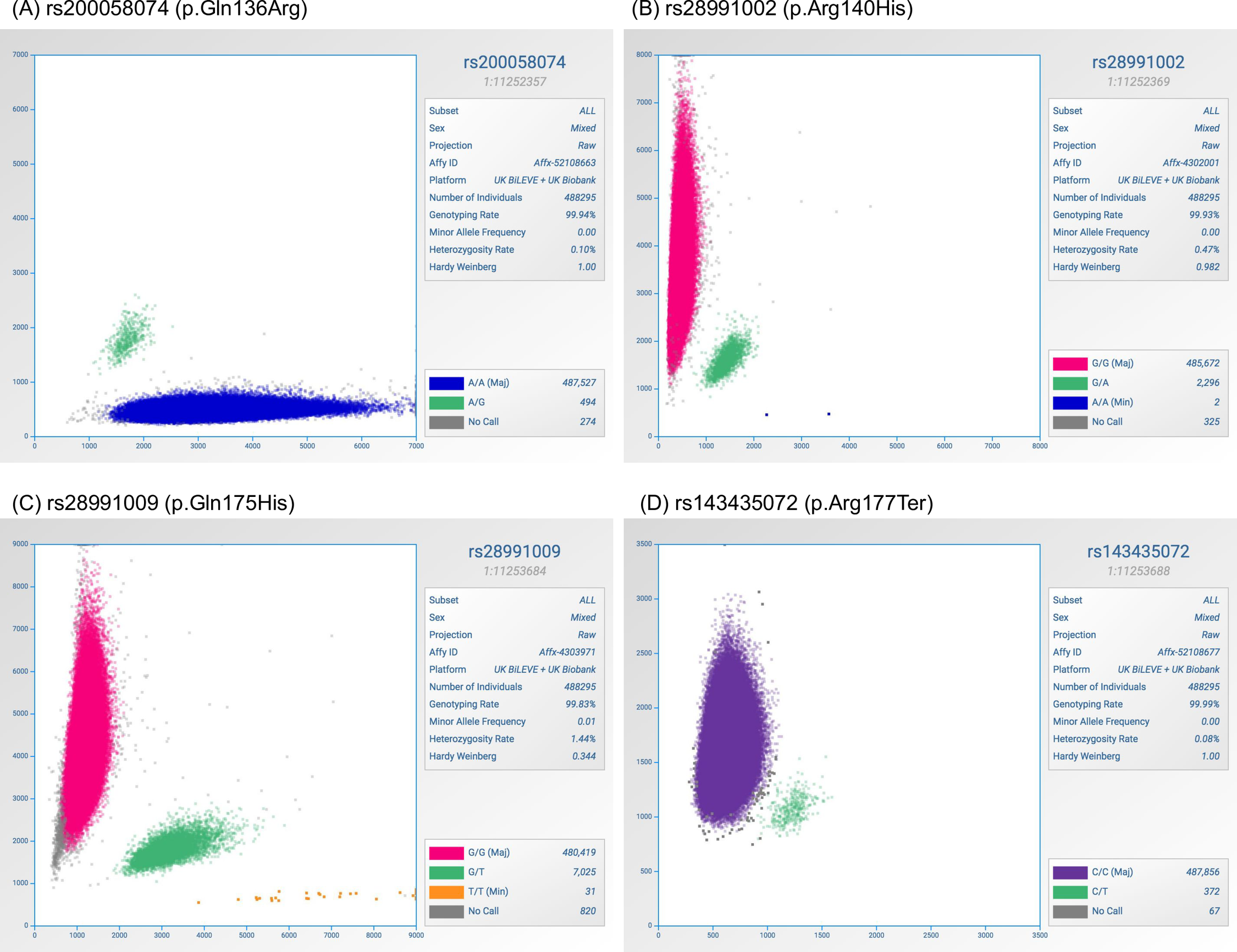

Supplement: S5 Fig — The intensity plots for ANGPTL7 protein-altering variants with 0.01% < MAF < 1%. (A) rs200058074 (p.Gln136Arg). (B) rs28991002 (p.Arg140His). (C) rs28991009 (p.Gln175His). (D) rs143435072 (p.Arg177Ter). (TIF) [file pgen.1008682.s006.tif]

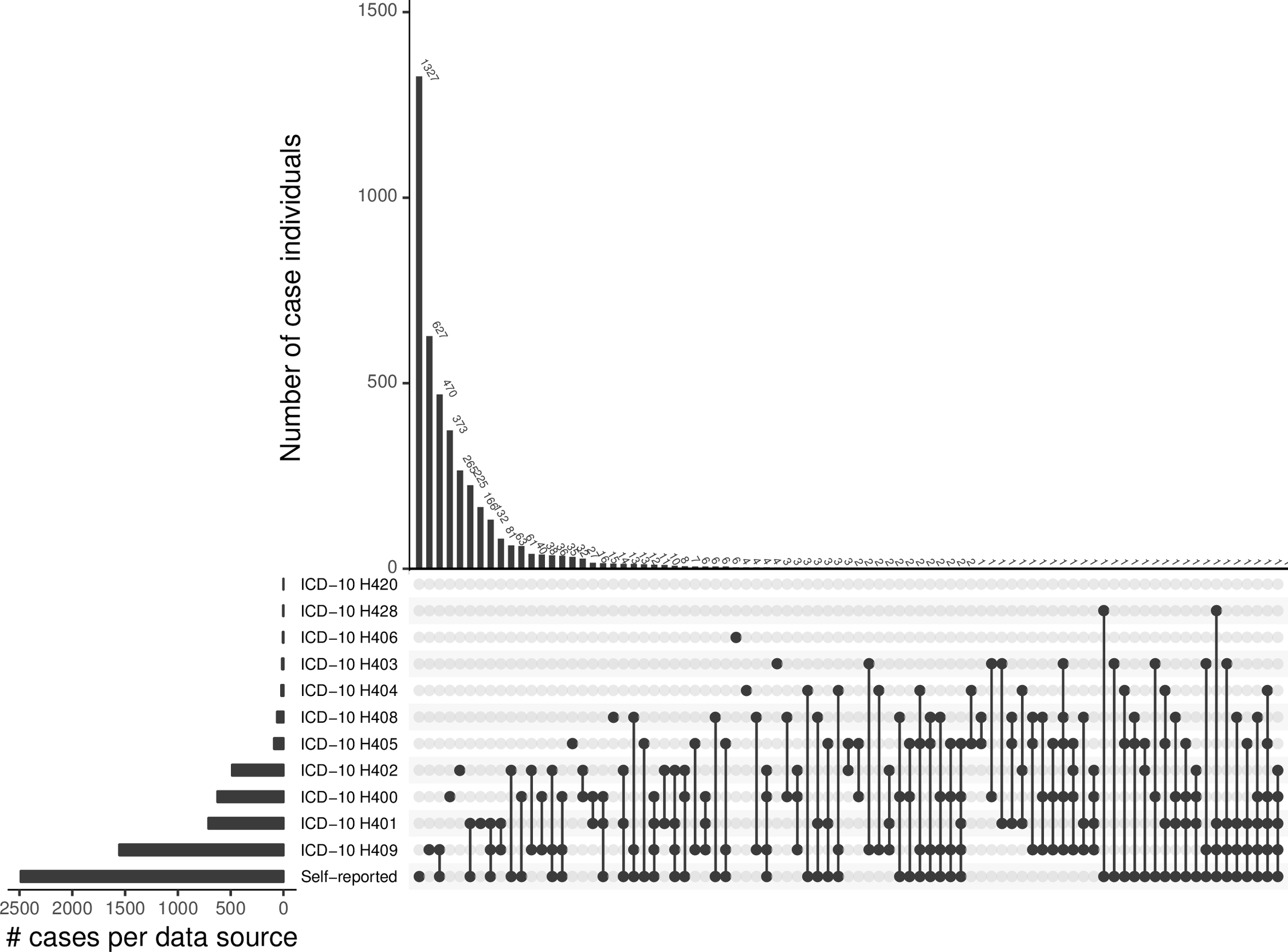

Supplement: S6 Fig — The breakdown of the data sources used for the definition of glaucoma in UK Biobank. The combination of self-reported glaucoma (coded as "1277" in UKB Data coding ID 6) and ICD-10 codes from hospital inpatient data are used for the glaucoma definition in UK Biobank. The number of individuals in the white British individuals without IOP measurements are shown. (TIF) [file pgen.1008682.s007.tif]

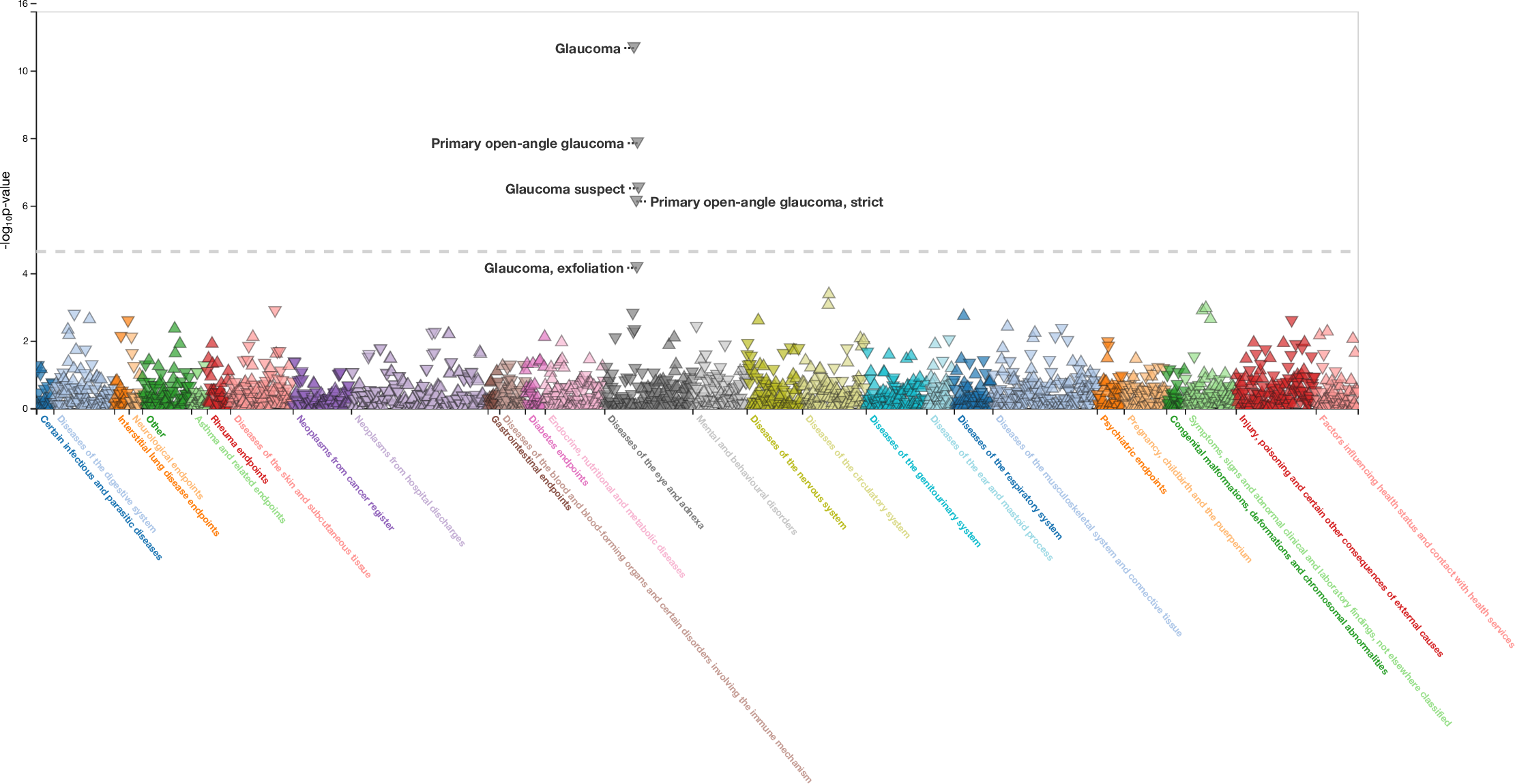

Supplement: S7 Fig — Phenome-wide association analysis of p.Arg220Cys in FinnGen. -log10(P-value) is displayed on the y-axis. Disease endpoints grouped by disease categories are displayed on the x-axis. Highlighted associations with P < 1x10^-4^ are shown. (TIF) [file pgen.1008682.s008.tif]

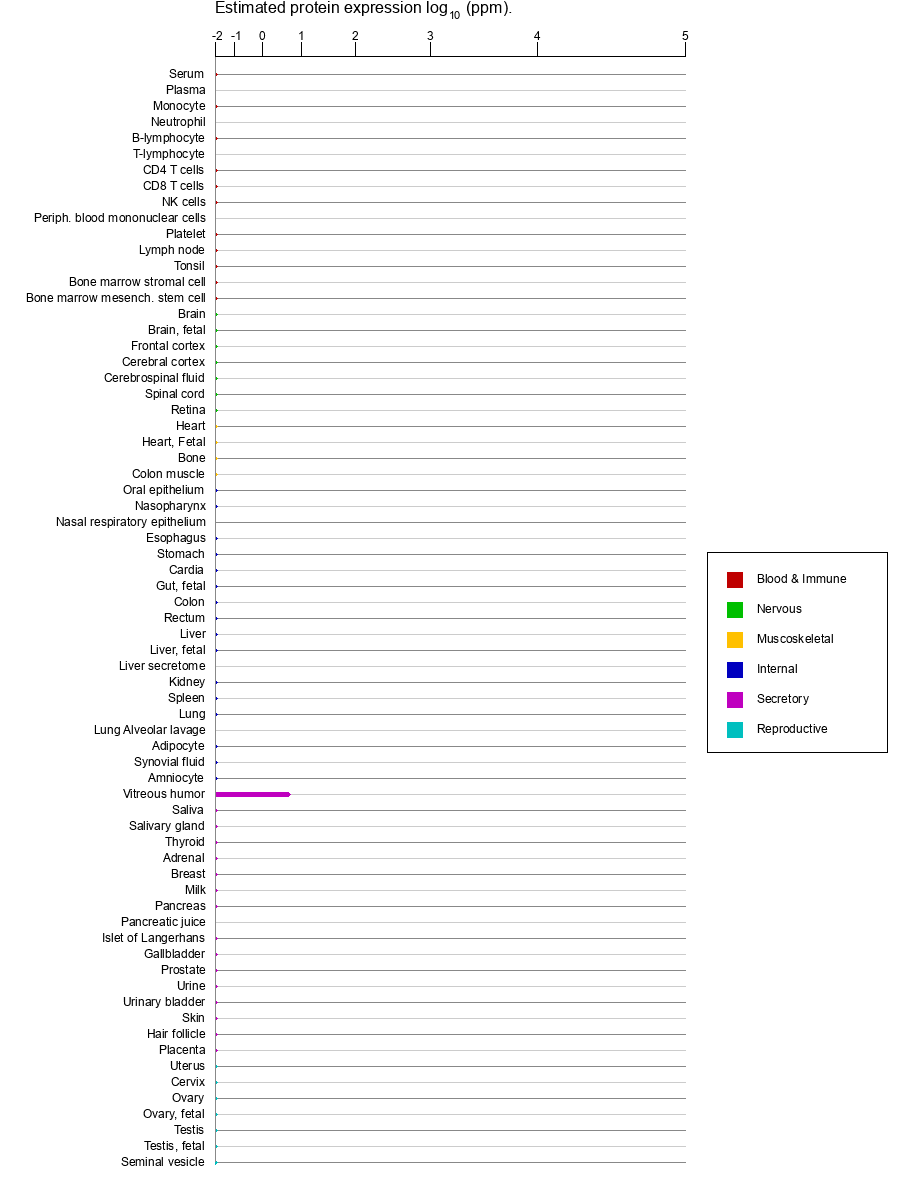

Supplement: S8 Fig — Protein expression in normal tissues and cell lines from ProteomicsDB and MOPED for ANGPTL7. (TIF) [file pgen.1008682.s009.tif]

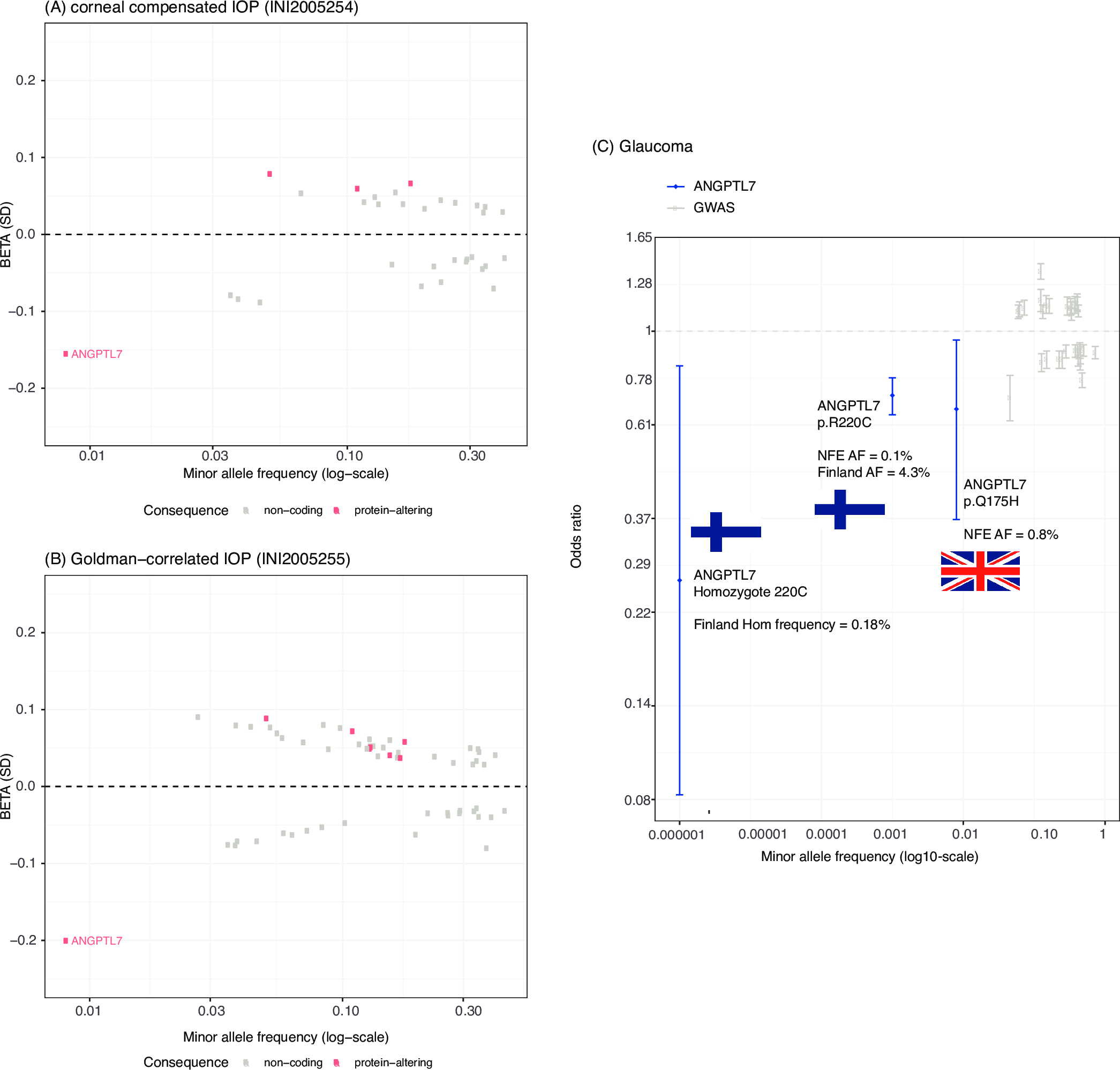

Supplement: S9 Fig — The cascade plot for corneal compensated (A) and Goldman-correlated (B) intraocular pressure association analysis in UK Biobank. The cascade plot for glaucoma (C) from published genome-wide significant GWAS associations (gray) and the variants highlighted in our paper. The minor allele frequency and the BETA (SD) are plotted for the LD-pruned variants with P < 5x10^-8^. The odds ratios are included for LD pruned published variants with P < 5x10^-8^ for glaucoma. (TIF) [file pgen.1008682.s010.tif]
