# Supplementary material for: Rare protein-altering variants in ANGPTL7 lower intraocular pressure and protect against glaucoma
Source: PLoS Genet. 2020 May 5;16(5):e1008682. doi: 10.1371/journal.pgen.1008682 (PMC7199928; doi:10.1371/journal.pgen.1008682)
Supplement: Attachment — Submitted filename: ANGPTL7_Glaucoma_review_response_20191218.pdf [file pgen.1008682.s024.pdf]

Department of Biomedical Data Science
AT STANFORD MEDICINE

Medical School Office Building
1265 Welch Road, 3rd floor
Stanford, CA 94305-5464
mrivas@stanford.edu
http://rivaslab.stanford.edu

12/18/2019

Dear Dr Bailey,

Please find the review response and revision regarding our manuscript “**Rare protein-altering variants in *ANGPTL7* lower intraocular pressure and protect against glaucoma**” (PGENETICS-D-19-01516). We thank the reviewers for their constructive comments and their time. We believe that the changes made in the light of their comments have significantly improved the manuscript.

Our responses to the reviewers below are in blue font, the comments from the reviewer are copied in black, and quoted texts from the updated manuscript are shown in gray with a vertical bar (examples are shown below):

This is an example of reviewer’s comments

This is an example of our response.

> *This is an example of quoted texts from the updated manuscript*

Reviewer #1

Reviewer #1: Primary open-angle glaucoma (POAG), and its endophenotypes, including intraocular pressure (IOP), are strongly heritable, and numerous risk loci have been identified through genomewide association studies (GWAS). The role of rare genetic variation, on the other hand, has received far less attention. This manuscript addresses the role of rare variants in IOP using two large cohorts, the UK Biobank and FinnGen. A single-variant association analysis of the UK Biobank sample revealed several nonsynonymous coding variants in ANGPTL7 that, on average, lower IOP. An independent missense variant in ANGPTL7 the FinnGen sample also reduces IOP, suggesting a general protective role of ANGPTL7 inactivation in POAG.

This study is of high potential impact, considering the large public health burden of POAG in the elderly and the paucity of knowledge of the contribution of rare genetic variation. A strong point of this article is the large sample size, suitable for studies of rare variants, and meticulous quality control and documentation of the analysis on the UK Biobank cohort. The manuscript is written fairly well, with a few typos and minor grammatical errors.

Thank you very much for taking the time to review the manuscript.

We are confident that your comments have improved the clarity of the manuscript.

However, the manuscript is marred by improperly conducted statistical analyses and lack of important information in the Methods and Discussion.

We apologize that the statistical analyses were not more clearly articulated in the earlier version of the manuscript. On a few comments including the connection between fixed-effects meta-analysis and burden test we would like to point the reviewer to Lee et al. AJHG 2013 that shows that the score test statistic for burden test assuming homogeneity of effects is given by

$$Q_{metaburden} = \left(\sum_{j=1}^{m} \sum_{k=1}^{K} w_{kj} S_{kj} \right)^2,$$

where j is an index for all variants in a study and k is an index for all K studies. Given that we are not analyzing across multiple studies then $K = 1$, and the meta-burden test is simply

$$Q_{metaburden} = \left(\sum_{j=1}^{m} w_j S_j \right)^2,$$

and with equal weights for the variants, it becomes a sum of statistics, which is analogous to a fixed-effects meta-analysis model. We have shown the equivalence of a burden test in a Bayesian setting in Deboever et al. 2018 (bioRxiv). However, we restrict to the frequentist

version. Given the equivalence, we have restricted our analysis to the “burden” and “dispersion” test framework and reported the results of that analysis here. We have also included a description in Methods along with reference to Moutsianas et al. 2015, PLoS Genetics that provides a detailed comparison of burden and dispersion style tests.

Specifically, a meta-analysis is inappropriate for measuring the aggregate effect of multiple genetic variants. A simple burden test would be more appropriate,

Please see above for description showing the equivalence between a fixed-effects meta-analysis using summary statistics to the burden test. Furthermore, see Liu et al. 2014 Nature Genetics:

“In Supplementary Notes, we show that rare variant statistics generated in this way are identical to those obtained by sharing individual-level data and allowing for heterogeneity in nuisance parameters, with no loss of power.” specifically the section titled “Burden Tests That Assume Variants Have Similar Effect Sizes”.

Given the confusion, we have updated the description of our method and results to only reflect p-value of a burden and dispersion test and have removed the effect size estimate summaries that may be obtained from a fixed-effects meta-analysis calculation. We now include the following text in Methods page 14, lines 404-414.

> *Burden and dispersion tests of rare protein-altering variants*
>
> *To assess associations with rare protein-altering variants, we performed a burden and dispersion test implemented in multiple rare variants and phenotypes (MRP) package with farebrother option (https://github.com/rivas-lab/ANGPTL7/tree/master/gene_based_test)[43,44]. The approach implemented in the MRP package is a generalization of the gene-based test for a single phenotype described in the Supplementary Material of Band et al. [45]Region-based test and subsection labeled calculating p-values. We used the GWAS summary statistics of rare (0.01 % < MAF < 1%) protein-altering variants characterized form the procedure above as the input data and performed the genome-wide burden and dispersion tests. The results of the burden and dispersion analysis are publicly available at NIH’s instance of figshare (Web Resources).*

as well as an estimate of the proportion of phenotypic variation explained by the variants.

Given that these are rare variants we expect that the proportion of phenotypic variation explained by the variants would be small and have large uncertainty estimates. However, we have applied GCTA’s GREML to the data to obtain an estimate.

On page 36, lines 810-815, we have included Supplementary Table S4 with the GCTA estimates of phenotypic variance explained by the rare variants in *ANGPTL7* for the IOP measures and

glaucoma. Furthermore, we have included in page 15, lines 431-436 of the main text the following sentence with the estimates:

> *Local heritability analysis*
>
> *To estimate the proportion of phenotypic variation explained by the rare protein-altering variants in ANGPTL7, we used Haseman-Elston (HE) regression using the cross product of the phenotypes for pairwise individuals implemented in genome-wide complex trait analysis (GCTA) version 1.92.4beta2[46,47]. We computed the genetic relationship matrix (GRM) using the 4 rare protein-altering variants in ANGPTL7 and used it for the HE regression analysis[48].*

If the authors feel that a meta-analysis is suitable here, they need to explain their rationale very carefully.

We have removed mentioning of the meta-analysis approach and listed the burden and dispersion test p-value results.

Moreover, an analysis of genetic correlation produced an impossible correlation estimate of 1.08, suggesting that some other quantity besides correlation is being measured. These serious errors cast doubt on the reliability of the main findings, even though the primary association analysis seems sound.

We thank the reviewer for this observation. Indeed, we used bivariate LD-score regression (Bulik-Sullivan 2015 Nat Gen) to estimate the correlation of genetic effects between the phenotypes using summary statistics. This method is an unbounded estimator as the contributor of the package acknowledges on the online discussion forum in the GitHub issue page.

https://github.com/bulik/ldsc/issues/89

> *LD score regression is not a bounded estimator, so it can produce estimates outside of [-1,1] due to sampling variation*

Estimates of genetic correlation above 1 have also been observed in previous publications including Table S5 of Ganna et al. 2019 Science, and UK biobank genetic correlation atlas from Neale's lab.

Given the concern of not having a bounded estimate of genetic correlation, which we agree is a valid concern, we have updated our genetic-correlation analysis using Bivariate Haseman-Elston (HE) regression implemented in GCTA, which allows us to estimate both the proportion of phenotypic variance explained by these rare variants and also the genetic correlation for the IOP measures analyzed in this study.

Please see Methods section pp 15 lines 437-441 for updated Methods description and main text pp 7 lines 84-87 for the inclusion of updated estimates.

> *Genetic correlation analysis*
>
> *To estimate the genetic correlation, we used bivariate-HEreg using the cross product of the phenotypes for pairwise individuals implemented in GCTA version 1.92.4beta2. We computed GRM based on non-rare (MAF > 1%) variants on the genotyping array and used it for the bivariate-HEreg analysis.*

> *These signals were consistently observed in corneal-compensated and Goldmann-correlated tonometry IOP measures for both left and right eyes (Supplementary Table S5), which is expected as the genetic correlation among those range from 0.75 to 1.0 (Supplementary Table S6).*

It is surprising that a genomewide gene-level association analysis of rare variants (e.g., burden test or SKAT), which would increase power to detect rare-variant aggregate effects, was not conducted.

We thank the reviewer for this suggestion. We have applied the burden and dispersion tests across all protein-altering rare variants in UK Biobank and reported the association results with the *ANGPTL7* signal. We restricted our analysis to variants with a minor allele frequency of less than 1%. Results from the analysis are now shown in Figure 1 and described in Methods, page 14, lines 404-414.

> *Burden and dispersion tests of rare protein-altering variants*
>
> *To assess associations with rare protein-altering variants, we performed a burden and dispersion test implemented in multiple rare variants and phenotypes (MRP) package with farebrother option (https://github.com/rivas-lab/ANGPTL7/tree/master/gene_based_test)[43,44]. The approach implemented in the MRP package is a generalization of the gene-based test for a single phenotype described in the Supplementary Material of Band et al. [45]Region-based test and subsection labeled calculating p-values. We used the GWAS summary statistics of rare (0.01 % < MAF < 1%) protein-altering variants characterized form the procedure above as the input data and performed the genome-wide burden and dispersion tests. The results of the burden and dispersion analysis are publicly available at NIH's instance of figshare (Web Resources).*

Methods for several analyses are missing, including the measurement of genetic correlation (Suppl. Fig 7) and the PheWAS (Suppl. Fig. 8).

We have included a methods description of the PheWAS, labelled "PheWAS analysis in UK Biobank" pp 15 lines 442-456 and "PheWAS analysis in FinnGen" pp 15 lines 457-487, and the genetic correlation (as described above). Furthermore, we provide a detailed table of the PheWAS results in Supplementary Table S10, Supplementary Data 2, Supplementary Data 3.

The PheWAS is only mentioned once in the main text, and only for citing the association result for Glaucoma; the significance of the other results from the PheWAS are not discussed.

Given that there is no other association, although the absence of association does not indicate the absence of genetic effects, we find it relevant to report the association statistics of those variants to those traits. To that end, we provide those statistics in the supplementary table for both UK Biobank and FinnGen in Supplementary Table S10, Supplementary Data 2, and Supplementary Data 3.

We have also included the following sentence on page 9 line 162-164 of the main text of the paper referencing the PheWAS results

> *Through phenome-wide association analysis (PheWAS), we did not find any significant association for non-eye phenotypes (P>1.0 x 10-5 for both in UK Biobank and FinnGen, Supplementary Table S10, Supplementary Data 2, Supplementary Data 3).*

The Discussion is perfunctory and is lacking important content. What are the limitations of the study? Specifically, how does the lack of overlap between POAG risk loci and IOP loci (e.g., Springelkamp et al., 2017, PMID 28073927) affect the significance of these results for treating POAG?

In the Discussion, we have added limitations of the study including:

1. Inability to assess whether the missense variants are partial loss-of-function variants, dominant negative, or gain of function given the data we have at hand.
2. Inability to identify the cell type where these variants are acting on.
3. Inability to assess whether the effect modifies progression of glaucoma (e.g. from diagnosis to potential surgery).
4. We have included references to Springelkamp et al.

For the question pertaining to the lack of overlap between POAG risk loci and IOP loci, in this study we have evidence that the variants in *ANGPTL7* impact IOP using UK Biobank data and have a protective effect on glaucoma risk, for example in FinnGen p.R220C has a protective effect on all glaucoma (number of cases n=6537, p=1.9e-12, OR=0.70), primary open-angle glaucoma (POAG, n=3357, OR=0.68), exfoliation glaucoma (n=1185, p=6.7e-5, OR=0.64), primary angle-closure glaucoma (n=466, p=1.6e-3, OR=0.59), and signal in normotensive glaucoma with limited sample size (n=653, p=0.07, OR=0.78). These subtype analyses are now included in Figure 1.

We included those points in Discussion (pp 10-11, lines 219-261)

When combined with the previously-reported associations with IOP and glaucoma, our results provide compelling genetic evidence of the role of ANGPTL7 in glaucoma and its subtypes including exfoliation, primary open-angle, and primary angle-closure, which may come in contrast to prior findings with lack of overlap between POAG risk and IOP loci[32]. In the context of the other established variants in glaucoma, including the protein-truncating variants in MYOC, p.Gln175His and the 57-fold Finnish-enriched p.Arg220Cys variant in ANGPTL7 exerts a comparable protective effect. While our genetic discovery provides compelling evidence of involvement of ANGPTL7 in glaucoma, several important questions remain to be answered before its eventual clinical translation. First, we were not able to assess whether the missense variants are complete loss-of-function, partial loss-of-function variants, dominant negative, or gain of function given the data we have at hand. Although we do have a predicted protein-truncating variant, p.Arg177Ter, with nominal evidence of association to IOP and an estimated effect consistent with the missense substitutions, it is challenging to draw conclusions about its functional consequence from in silico predictions, as we have reported in earlier studies assessing when PTVs trigger degradation pathways like nonsense-mediated decay[33]. Second, it is unclear in which cell types these variants are acting on to confer the protective and IOP lowering effects. We anticipate that ANGPTL7 may be acting in the trabecular meshwork given its high expression in both adult and fetal trabecular meshwork (> 3000 FPKM)[34], we see high expression in both adult and fetal cornea (>200 FPKM), which introduces some challenges as how we interpret its functional role, and we hypothesize that given its high expression in cornea it may be one reason why we see stronger evidence of association in IOP Goldman correlated measures compared to corneal compensated IOP. Additionally, future studies should assess whether ANGPTL7 variants modify the progression of glaucoma, for example, whether ANGPTL7 carriers are less likely to go from glaucoma diagnosis to potential surgery. Although we are aggregating these data, we are thus far unable to draw definitive conclusions.

Because of the strong protective effect associated with the ANGPTL7 protein-altering variants (Supplementary Figure S9), further studies of ANGPTL7 inhibition and the specific action of these variant proteins should be useful in understanding the mechanism by which glaucoma protection occurs and whether this reveals a promising therapeutic opportunity similar to that which has been realized from the examples of PCSK9, APOC3 and cardiovascular disease[35–37]. Given the rapidly evolving field of gene editing and siRNA, we can only speculate that if the effect is truly loss-of-function and that gene inhibition is an appropriate strategy then these therapeutic modalities will be especially relevant. Therapeutic delivery is also a complicated challenge. Although injection to the eye is currently commonplace in practice, it is unclear whether different therapeutic modalities, e.g. antibody, siRNA, CRISPR, base-editing would be appropriate, and whether the duration of the treatment would be sufficiently durable to be effective to prevent extremely frequent injections or competitive against current therapeutic modalities. New drug delivery technologies are of interest and it is clear that a durable and efficient mode of delivery that mimics the protective effect of these mutations is an attractive strategy. Our genetic data from ANGPTL7 homozygotes with up to a 69% risk reduction for all glaucoma and 80% risk reduction for primary open-angle glaucoma suggest that this is likely to be a safe and effective strategy for therapeutic intervention.

What are the implications of the differences in allele frequency of IOP-lowering variants in the Finnish and UK populations?

In the Discussion (pp 9-10, lines 190-208) we have included text summarizing the implications of the differences in allele frequency of IOP-lowering variants in the Finnish and UK populations. More specifically, that the enrichment of the 220C allele in the Finnish population enables future recall studies and the potential to recall individuals homozygote for the allele, which can eventually improve our understanding of the mechanism by which *ANGPTL7* disruption leads to protection to glaucoma risk and lowering of IOP. Furthermore, this is evidence that the bottleneck property of Finland enables powerful gene discovery and replication of independent set of alleles to increase our confidence in the gene as it provides a convincing allelic series.

This study establishes strong genetic evidence for the involvement of ANGPTL7 in glaucoma risk in which a powerful allelic series, including multiple low-frequency missense substitutions and a single premature stop-gain substitution, is conclusively associated with reduced disease risk and endophenotype-lowering effects. Our results highlight the benefit of rare protein-altering variant analysis using multiple large cohorts, especially when the population history of the participating cohort experienced a bottleneck, which enables an enrichment of rare alleles as we report with the ANGPTL7 p.Arg220Cyc allele[13]. In Finland, the most common glaucoma subtypes are POAG and the secondary exfoliation glaucoma. The main difference in glaucoma prevalence is that in Finland the exfoliation glaucoma is much more prevalent (31%) than in the UK[22]. The prevalence of POAG is similar in Finland than in other European populations. The prevalence is heavily affected by age. In one Finnish cohort study, among individuals aged 70 years or older, the prevalence of POAG was approximately 7%[23]. Relative similar prevalence for POAG is reported in European populations[24]. Many patients with POAG are undiagnosed so the prevalence is affected by sampling methods (i.e. cohort or diagnosis reported). The population cohorts from founder populations enables future recall studies focusing on individuals homozygous for the allele, which can eventually improve our understanding of the mechanism by which ANGPTL7 disruption leads to protection to glaucoma risk and lowering of IOP. The discovery of two independent protein-altering alleles with directionally consistent effects from the two analyzed populations increase our confidence in the gene's causal link to glaucoma.

How do the new findings for ANGPTL7 fit in with what is already known about its role in POAG and IOP?

IOP is the main risk factor for glaucoma and the only one which can be affected by treatments (medication, laser, and surgery). The ANGPTL7 protein is expressed in the trabecular meshwork and preliminary experimental data suggest that it affects the IOP level. Similarly, a well-known risk gene for POAG, the *MYOC* gene, affects IOP in the trabecular meshwork. Naturally, more experimental work is needed to elucidate the precise role of ANGPTL7 in the regulation of IOP for glaucoma risk. Our data suggest that at least partly ANGPTL7 glaucoma risk work through IOP. In the Discussion (pp 10-11, lines 213-244) we have added the following text to clarify:

ANGPTL7 overexpression in primary human trabecular meshwork cells was found to alter the expression of relevant trabecular meshwork proteins of the extracellular matrix, including fibronectin, collagens type I, IV, and V, myocilin, versican, and MMP1, and ANGPTL7 protein was increased as the disease progressed in POAG beagle dogs[17]. The tissue-specific protein expression data suggest that further work in dissecting the role of ANGPL7 in all possible cell types in the eye is warranted.

When combined with the previously-reported associations with IOP and glaucoma, our results provide a compelling genetic evidence of the role of ANGPTL7 in glaucoma and its subtypes including exfoliation, primary open-angle, and primary angle-closure, which may come in contrast to prior findings with lack of overlap between POAG risk and IOP loci[28]. In the context of the other established variants in glaucoma, including the protein-truncating variants in MYOC, p.Gln175His and the 57-fold Finnish-enriched p.Arg220Cys variant in ANGPTL7 exert a comparable protective effect. While our genetic discovery provides a compelling evidence of involvement of ANGPTL7 in glaucoma, several important questions remain to be answered before its eventual clinical translation.

…

Second, it is unclear in which cell types these variants are acting on to confer the protective and IOP lowering effects. We anticipate that ANGPTL7 may be acting in the trabecular meshwork given its high expression in both adult and fetal trabecular meshwork (> 3000 FPKM)[30], we see high expression in both adult and fetal cornea (>200 FPKM), which introduces some challenges as how we interpret its functional role, and we hypothesize that given its high expression in cornea it may be one reason why we see stronger evidence of association in IOP Goldman correlated measures compared to corneal compensated IOP. Additionally, future studies should assess whether ANGPTL7 variants modify the progression of glaucoma, for example whether ANGPTL7 carriers are less likely to go from glaucoma diagnosis to potential surgery. Although we are aggregating these data, we are thus far unable to draw definitive conclusions.

Specific comments:

1. Introduction, p. 2: Khawaja et al. (ref. 6) alone identified 68 risk loci for IOP. The total number of risk loci, including previous studies, including is larger. Choquet et al (2018) PMID 29235454 and Hysi et al. (2014) PMID 25173106 should also be cited,

Thank you. We've included those citations and updated the text in pp 3 lines 63-65 to reflect the updated number of risk loci for IOP. We note, however, that only a subset are associated with glaucoma at genome-wide significance ($P < 5x10^{-8}$).

> *More than 68 independent loci have been implicated in IOP by meeting the GWAS significance threshold of association ($P < 5x10^{-8}$) [5–8].*

The term “unequivocally implicated” should be made clearer: does this mean replicated within one study? between two or more independent studies?

Thank you. We’ve included a description to accurately reflect that this corresponds to the GWAS significance threshold of association as described above in the quoted text.

2. Results, p. 3: How was the joint association analysis for the three less significant ANGPTL7 SNPs performed? By a burden test?

Yes, we have applied a burden test and reported the p-value. The description of the Methods has also been updated in the Section titled *Burden and dispersion tests of rare protein-altering variants* in pp 14, lines 404-414.

3. Results p. 4: The lack of significance in the associations with glaucoma may also be explained by misclassification in the glaucoma phenotype on account of its being based on EHR, and by the likely presence of normal-tension POAG patients within the glaucoma cases.

Indeed. However, the three additional rare protein-altering variants (removing p.Gln175His) have a composite allele frequency of 0.345%. This is consistent with power calculations, using Genetic Power Calculator (http://zzz.bwh.harvard.edu/gpc/), where our power to detect association for a binary trait in 4,238 cases and 250,660 controls at alpha = 0.05, and 0.001, i.e. P < .05 and .001, with OR of 0.7 (30% reduction in risk) is equal to 62% and 15.2%, respectively. This has also been included in the main text pp 7-8, lines 178-181.

> *This is consistent with power calculations, using Genetic Power Calculator[15], where our power to detect association for rare variants with a composite allele frequency of 0.345% and a binary trait in 4,238 cases and 250,660 controls at alpha = 0.05, and 0.001, i.e. P < .05 and .001, with OR of 0.7 (30% reduction in risk) is equal to 62% and 15.2%, respectively.*

4. Results, pp. 5-6. The last paragraph of the Results belongs in the Discussion, except for the sentence on tissue-specific expression of ANGPTL7.

We thank the reviewer and have now moved the last paragraph of the Results section in the Discussion and included the sentence on the tissue-specific expression of ANGPTL7 in the Results text.

5. Discussion: Does the Finnish population have a different prevalence of POAG than the UK?

We’d like to thank the reviewer for this relevant question. In Finland, the most common glaucoma subtypes are POAG and the secondary exfoliation glaucoma. The main difference in glaucoma prevalence is that in Finland the exfoliation glaucoma is much more prevalent (31%) than in the UK (PMID: 18435818). The prevalence of POAG is similar in Finland than in other

European populations. The prevalence is heavily affected by age. In one Finnish cohort study, among individuals aged 70 years or older, the prevalence of POAG was approximately 7% (PMID: 7607812). Relative similar prevalence for POAG is reported in European populations (PMID: 24974815). Many patients with POAG are undiagnosed so the prevalence is affected by sampling methods (i.e. cohort or diagnosis reported).

We have added the three references indicated above and have also included the following text in the Discussion (pp 9-10, lines 245-263):

> *This study establishes strong genetic evidence for the involvement of ANGPTL7 in glaucoma risk in which a powerful allelic series, including multiple low-frequency missense substitutions and a single premature stop-gain substitution, is conclusively associated with reduced disease risk and endophenotype-lowering effects. Our results highlight the benefit of rare protein-altering variant analysis using multiple large cohorts, especially when the population history of the participating cohort experienced a bottleneck, which enables an enrichment of rare alleles as we report with the ANGPTL7 p.Arg220Cyc allele[13]. In Finland, the most common glaucoma subtypes are POAG and the secondary exfoliation glaucoma. The main difference in glaucoma prevalence is that in Finland the exfoliation glaucoma is much more prevalent (31%) than in the UK[22]. The prevalence of POAG is similar in Finland than in other European populations. The prevalence is heavily affected by age. In one Finnish cohort study, among individuals aged 70 years or older, the prevalence of POAG was approximately 7%[23]. Relative similar prevalence for POAG is reported in European populations[24]. Many patients with POAG are undiagnosed so the prevalence is affected by sampling methods (i.e. cohort or diagnosis reported). The population cohorts from founder populations enables future recall studies focusing on individuals homozygous for the allele, which can eventually improve our understanding of the mechanism by which ANGPTL7 disruption leads to protection to glaucoma risk and lowering of IOP. The discovery of two independent protein-altering alleles with directionally consistent effects from the two analyzed populations increase our confidence in the gene's causal link to glaucoma.*

6. Methods: Was the entire age range of the UK Biobank dataset included? The genetic determinants of IOP before age 40 may well be different than in older individuals. Was the average age of rare-variant carrying individuals much different from that of the entire sample?

Thank you for the question. Yes, the entire age range of the UK Biobank cohort is included (we have specified this in the Methods section now pp. 12 and lines 349-350).

> *Of note, we included the entire age range of the UK Biobank cohort for our analysis to maximize the power of association analysis.*

Here is the age distribution stratified by the *ANGPTL7* protein-altering variant (p.Gln175His, rs28991009) carriers in UK Biobank. The KS test comparing the carriers (G/G or G/T) vs.

non-carriers (T/T) showed that the difference in the age distributions was not significant (p-value = 0.77).

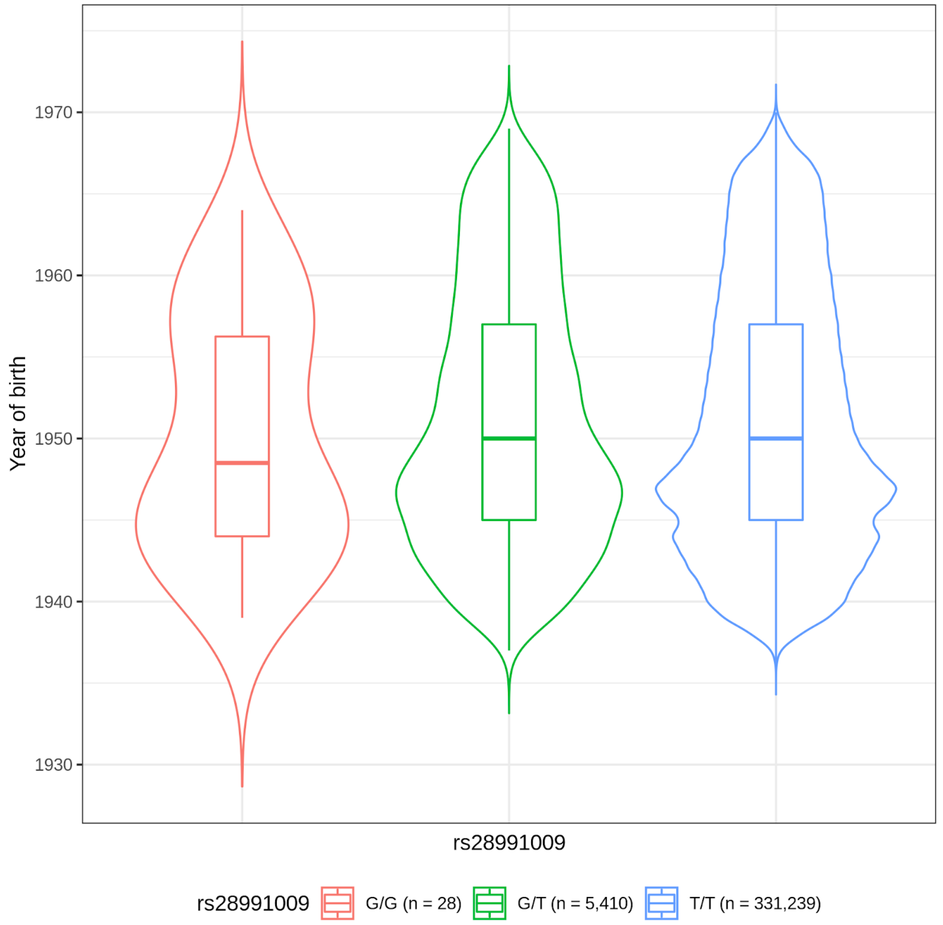

In our genetic analysis, we included age as a covariate in the model. This is now clarified in our manuscript in pp. 12-13 lines 359-372.

Rare protein-altering variant genome-wide association scan for IOP

For the white British individuals (n = 337,151) in UK Biobank[11], we applied genome-wide association analysis for directly genotyped variants and phenotypes with inverse-normal transformation (--pheno-quantile-normalize option) using generalized linear regression model implemented in PLINK v2.00aLM (12 Nov. 2019) with age, sex, types of genotyping array, and the first 4 genotype principal components, where array is an indicator variable that indicates whether the individual was genotyped using the UK BiLEVE array or the UK Biobank array, as described elsewhere[38,39]. The inverse-normal transformation (--pheno-quantile-normalize option in PLINK2) is a non-parametric phenotype normalization procedure and it forces the phenotype to a standard normal distribution, preserving just the quantiles. For example, if the original phenotype values are 9, 4, 9, and 7 in that order, the quantiles are 0.75, 0.125, 0.75, 0.375, and the transformed phenotype values are the inverse-normal-cdf of each of the quantile value[cite: https://www.cog-genomics.org/plink/2.0/data#quantile_normalize]. The genome-wide association summary statistics are available at NIH's instance of figshare (Web Resources).

7. Methods, p. 8 top: What is the "Array" predictor in the logistic regression model?

In the UK Biobank genotyping experiment two arrays were used: 1. UK BiLEVE array (49,950 individuals in the entire cohort, of which 37,036 individuals are in the unrelated White British individuals used in this study), and 2) UK Biobank axiom array (438,427 individuals in the entire cohort, of which 300,115 individuals are in the studied cohort). We have included it as a covariate as it may induce spurious association and we would like to adjust for any confounding that this may lead to.

We agree that the original text was not clear about this. We have updated the main text (pp. 12-13 lines 359-366.

Rare protein-altering variant genome-wide association scan for IOP

For the white British individuals (n = 337,151) in UK Biobank[11], we applied genome-wide association analysis for directly genotyped variants and phenotypes with inverse-normal transformation (--pheno-quantile-normalize option) using generalized linear regression model implemented in PLINK v2.00aLM (12 Nov. 2019) with age, sex, types of genotyping array, and the first 4 genotype principal components, where array is an indicator variable that indicates whether the individual was genotyped using the UK BiLEVE array or the UK Biobank array, as described elsewhere[38,39].

8. Suppl. Fig. S1: Considering the very large sample size, a density plot comparing IOP in cases and controls will provide more information than a boxplot. See Fig. 4 of Martin et al. (2017) PMID 28366442 for an example of overlapping density distributions.

Thank you for making this recommendation. We added the violin plot on top of the boxplot so that people can read the distribution (Supplementary Figure S1, quoted below).

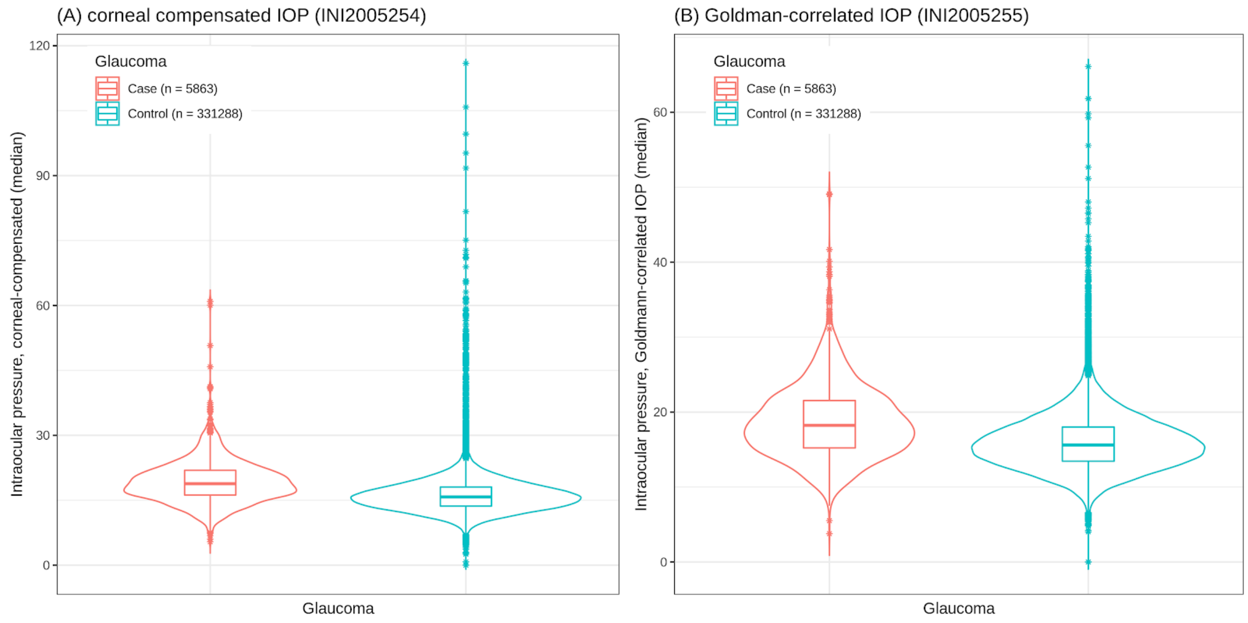

9. Suppl. Fig. S7: This information would be much more concisely shown in a table, or even in the text. It is not clear what the correlations are between: the three x-axis labels each mention only one variable.

We agree with the reviewer. We have changed Suppl. Fig. S7 to a table (quoted below).

Supplementary Table S6. Pairwise genetic correlation of IOP phenotypes. The genetic correlation (rg) for pairs of traits (Trait 1 and Trait 2, shown as GBE ID for 6 IOP traits (corneal compensated IOP [median INI2005254, right: INI5254, and left: INI5262] and Goldman-correlated IOP [median INI2005255, right: INI5255, and left: INI5263]) is shown with the standard error estimates (SE) based on Jackknife.

Trait 1	Trait 2	rg	SE
INI2005254	INI2005255	0.821284	0.0212266
INI2005254	INI5254	0.985296	0.0434097
INI2005254	INI5255	0.811856	0.0406103
INI2005254	INI5262	0.998911	0.0434669
INI2005254	INI5263	0.835363	0.039526
INI2005255	INI5254	0.798462	0.039117
INI2005255	INI5255	1.0073	0.0258203
INI2005255	INI5262	0.75938	0.043236

INI2005255	INI5263	0.993736	0.0259505
INI5254	INI5255	0.824087	0.0251798
INI5254	INI5262	0.984819	0.0567066
INI5254	INI5263	0.858557	0.0458405
INI5255	INI5262	0.748673	0.0485872
INI5255	INI5263	0.990019	0.0319751
INI5262	INI5263	0.803338	0.0292724

10. Suppl. Fig. S8: This PheWAS analysis isn't mentioned anywhere in the text, except to indicate that the association of R220C with glaucoma was highly significant. Can it be omitted from the paper except for the glaucoma-related phenotypes?

We have included a reference to the PheWAS analysis in the main text and included specific mention of the glaucoma-related phenotypes in pp 9 lines 234-238 . Furthermore, we have included the data as Supplementary Table S10, Supplementary Data 2, and Supplementary Data 3.

> *Through phenome-wide association analysis (PheWAS), we did not find any significant association for non-eye phenotypes (P>1.0 x 10-5 for both in UK Biobank and FinnGen, Supplementary Table S10, Supplementary Data 2, Supplementary Data 3). Hence, we did not find any severe medical consequences that would be of obvious concern in developing a therapeutic to mimic the effect of these alleles..*

11. Suppl. Table S1: The data would be more straightforward to interpret in the form of a small table for each variant with counts for each genotype pair observed (including NA), rather than one large table with the counts in a single column.

We agree with the reviewer. We have changed the data in the form of a small table for each variant with counts for each genotype pair observed (including NA). The updated tables are now presented as Supplementary Table S2 in page 34.

12. Suppl. Table S6: This appears to be raw, unformatted output, and should be formatted as a table.

We apologize. We have now changed it to be formatted with a table and is now Supplementary Table S8 in page 40.

Reviewer #2

Reviewer #2: This is an interesting study examining rare variant associations with glaucoma and its major endophenotype, IOP. There are several issue that need addressing.

Major comments:

- It is an odd approach to take Goldmann-correlated IOP of the right eye as a primary measure, and then not display results for the left eye measure or the corneal-compensated measures (only show genetic correlations). Are the authors hypothesizing that genetic associations with IOP may only influence one eye and not the author? If not, a better approach is to include both eyes and adjust for the correlation using a random-effects approach, or to simply take the mean of right and left eye measures.

As the reviewer suggested we have taken the median of all eye measures for a given individual and that phenotype is used as the default throughout the main text analyses. Using one eye may be better than incorporating both eyes because they are not independent of each other. This may be different for exfoliation glaucoma, but we do not have information of the eye affected by exfoliation if we were to assess the effect of *ANGPTL7* carriers on IOP in that setting. We have also included results for left eye measure and the corneal compensated measures per eye and combined (median) in Supplementary Table S5.

- Why is the primary analysis for Goldmann-correlated IOP? Corneal compensated IOP has been shown to be more reflective of true physiological IOP, and less influenced by corneal artefact. Could ANGPTL7 variation actually be influencing the cornea rather than IOP?

As the reviewer suggested we have also included the corneal-compensated IOP results and find a significant association with IOP as well ($P = 5.96 \times 10^{-9}$ and 1.07×10^{-13}, $\beta = -0.20$, 95% CI: [-0.21, -0.10]). We have included the corneal compensated IOP results in the text alongside with the Goldman-correlated results as shown in Figure 1. The association is significant in both Goldmann-correlated IOP and corneal compensated IOP. We note that *ANGPTL7* has high expression in both trabecular meshwork and cornea (adult and fetal, FPKM > 200) as described in the Discussion and it may be possible that one reason we are seeing more significant associations in Goldman-correlated IOP is that *ANGPTL7* is affecting IOP via both cornea-dependent and independent mechanisms.

- Were the IOP variables cleaned prior to analysis? If so, how?

We thank the reviewer for the comment. Yes, all of the IOP variables were inverse normalized across the UK Biobank cohort to ensure normality of the trait and adjusted for covariates including age, sex, and principal components. The normalization procedure is now described in pp. 12-13 lines 359-372.

Rare protein-altering variant genome-wide association scan for IOP

For the white British individuals (n = 337,151) in UK Biobank[11], we applied genome-wide association analysis for directly genotyped variants and phenotypes with inverse-normal transformation (--pheno-quantile-normalize option) using generalized linear regression model implemented in PLINK v2.00aLM (12 Nov. 2019) with age, sex, types of genotyping array, and the first 4 genotype principal components, where array is an indicator variable that indicates whether the individual was genotyped using the UK BiLEVE array or the UK Biobank array, as described elsewhere[38,39]. The inverse-normal transformation (--pheno-quantile-normalize option in PLINK2) is a non-parametric phenotype normalization procedure and it forces the phenotype to a standard normal distribution, preserving just the quantiles. For example, if the original phenotype values are 9, 4, 9, and 7 in that order, the quantiles are 0.75, 0.125, 0.75, 0.375, and the transformed phenotype values are the inverse-normal-cdf of each of the quantile value[cite: https://www.cog-genomics.org/plink/2.0/data#quantile_normalize]. The genome-wide association summary statistics are available at NIH's instance of figshare (Web Resources).

- What does the meta-analyzed effect estimate mean when combining effects at multiple different variants (Supp Tables 6 and 9)? Is this the effect you would expect to see if someone had all these variants together? It seems odd to me that you would search for IOP-lowering variants in a gene, and then meta-analyze these selected variant effects together. Surely this is biased and misleading? Unless the authors can make a very strong rationale, I would remove sections on "combined significance".

We'd like to thank the reviewer for the comment. Although meta-analysis of summary statistics is appropriate in this scenario, due to the equivalence between a burden test using individual-level data and fixed-effects meta-analysis using summary statistics (see references in the response to reviewer 1, who also raised this concern), we realize this would be a point of confusion for many readers. As a result, we have now updated the description of our method to reflect that we are applying burden and dispersion tests and report only the p-value associated with the analysis (page 14, lines 404-414).

Burden and dispersion tests of rare protein-altering variants

To assess associations with rare protein-altering variants, we performed a burden and dispersion test implemented in multiple rare variants and phenotypes (MRP) package with farebrother option (https://github.com/rivas-lab/ANGPTL7/tree/master/gene_based_test)[43,44]. The approach implemented in the MRP package is a generalization of the gene-based test for a single phenotype described in the Supplementary Material of Band et al. [45]Region-based test and subsection labeled calculating p-values. We used the GWAS summary statistics of rare (0.01 % < MAF < 1%) protein-altering variants characterized form the procedure above as the input data and performed the genome-wide burden and dispersion tests. The results of the burden and dispersion analysis are publicly available at NIH's instance of figshare (Web Resources).

- The definition of glaucoma in UK Biobank, a major outcome variable in the paper, is not clear. How many were identified using self-report? How many by hospital episode statistics? Why did the authors not limit to POAG HES codes?

We'd like to thank the reviewer for this comment. Indeed, the UK Biobank cohort has a mixture of definitions for which you can derive a phenotype from including self-reported data and hospital episode statistics. We updated Methods pp 13 lines 373-382 and Supplementary Figure S6 that provides a detailed breakdown of the definition of glaucoma in UK Biobank.

Glaucoma association analysis in individuals without IOP measurements

To assess the potential effects of identified putative IOP-lowering genetic variants on glaucoma risk, we applied the genome-wide association analysis for glaucoma (Global Biobank Engine phenotype ID: HC276) focusing on 254,898 individuals (4,238 cases and 250,660 controls) in UK Biobank who do not have any of the IOP measurements (Figure 1). The glaucoma phenotype was previously defined as a part of "high confidence" disease outcome phenotypes by combining disease diagnoses (UK Biobank Field ID 41202, 41204, 40001, and 40002) from the UK National Health Service Hospital Episode Statistics (ICD10 codes: H40.[0-6,8,9], H42.8, and Q15.0) with self-reported non-cancer diagnosis questionnaire (UK Biobank Field ID 20002), as summarized as an UpSet plot in Supplementary Figure S9 [11,12,40].

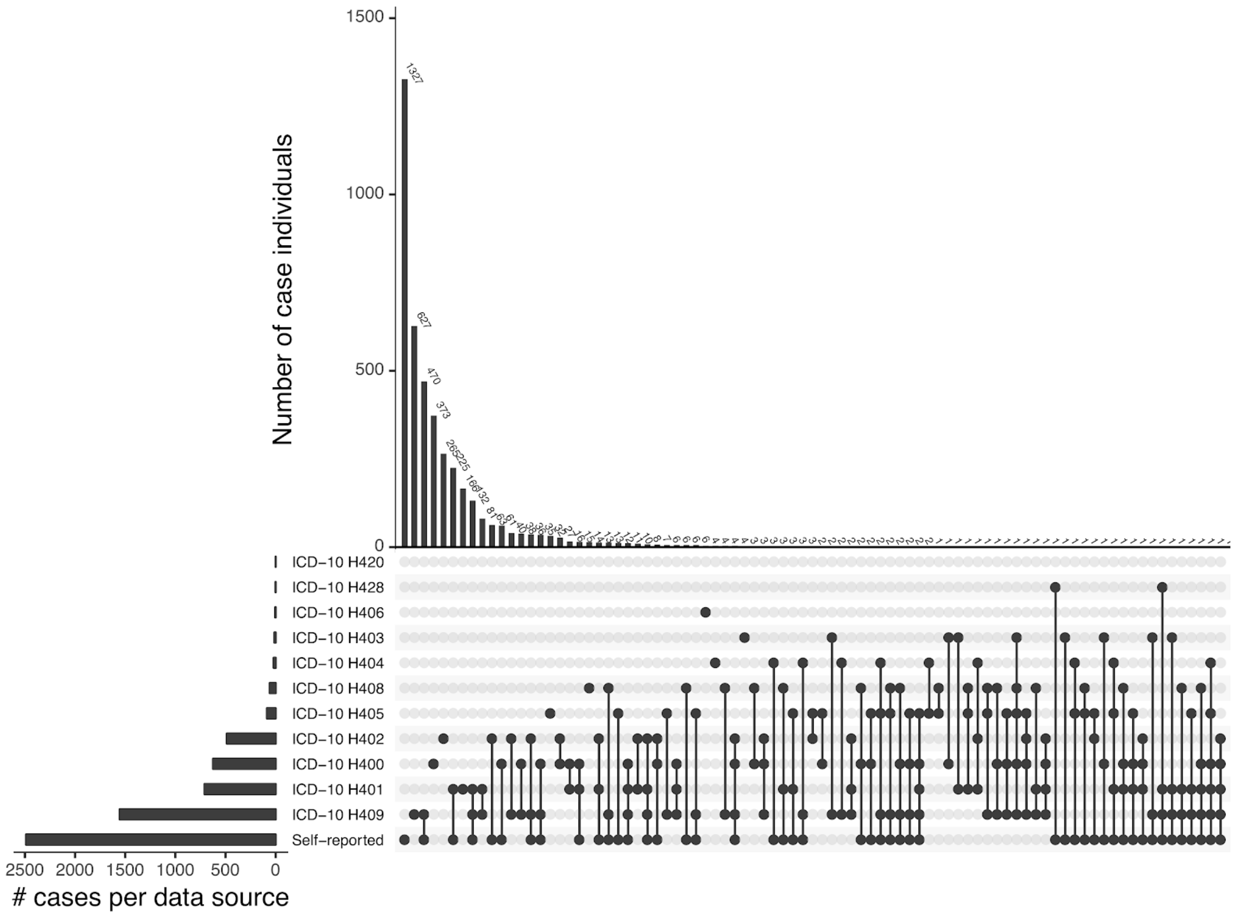

Supplementary Figure S6. The breakdown of the data sources used for the definition of glaucoma in UK Biobank. The combination of self-reported glaucoma (coded as "1277" in UKB Data coding ID 6) and ICD-10 codes from hospital inpatient data are used for the glaucoma definition in UK Biobank. The number of individuals in the white British individuals without IOP measurements are shown.

Furthermore, we find that the combination does improve power as we have shown for other diseases see Deboever et al. 2019 “Assessing digital phenotyping to enhance genetic studies of human diseases” (https://doi.org/10.1101/738856). In the FinnGen study, we report association, where most of our power exists given the higher frequency, to all glaucoma, POAG, and its subtypes.

How were controls defined, given that the glaucoma question was not administered to the whole cohort? Given this is a key outcome variable, I would recommend the authors present a flow chart for derivation of glaucoma status as well as IOP.

We would like to thank the reviewer for this suggestion. We have included a breakdown of the number of individuals for the derivation of glaucoma status (Supplementary Figure S6).

- Is it possible that the protein alteration increases function of the gene? What evidence do the authors have that the functional consequence of the identified variants is reduced gene function? Unless strong, the authors should temper the strength of the language they use to describe the effect.

We agree with the reviewer that this is a very important question, i.e. thus far we have not shown nor have data to suggest that the protein alteration either decreases or increases the function of the gene. However, we do have a predicted protein-truncating variant that is predicted to trigger nonsense-mediated decay that is likely to lead to loss of *ANGPTL7* function. Nonetheless, as the reviewer stated and as we have found (see Rivas et al. 2015 PMID:25954003), a large fraction of PTVs with triggering NMD prediction don't have data to support the presence of NMD degradation. As a result, we have tempered the strength of the language in the paper and have also added text in the Discussion clearly stating the limitations of the study and potential next steps that may help in improving our understanding of the functional consequences of these variants (page 10, lines 280-288).

> *While our genetic discovery provides compelling evidence of involvement of ANGPTL7 in glaucoma, several important questions remain to be answered before its eventual clinical translation. First, we were not able to assess whether the missense variants are complete loss-of-function, partial loss-of-function variants, dominant negative, or gain of function given the data we have at hand. Although we do have a predicted protein-truncating variant, p.Arg177Ter, with nominal evidence of association to IOP and an estimated effect consistent with the missense substitutions, it is challenging to draw conclusions about its functional consequence from in silico predictions, as we have reported in earlier studies assessing when PTVs trigger degradation pathways like nonsense-mediated decay[33].*

- The discussion is disappointingly short. How does this finding sit with other genetic discoveries for IOP and glaucoma? How does this fit in with what is known about IOP-related anatomy and physiology?

We would like to thank the reviewer for raising this point. We have included a section on the limitations of the current paper (pages 10-11, lines 280-299).

> *While our genetic discovery provides compelling evidence of involvement of ANGPTL7 in glaucoma, several important questions remain to be answered before its eventual clinical translation. First, we were not able to assess whether the missense variants are complete loss-of-function, partial loss-of-function variants, dominant negative, or gain of function given the data we have at hand. Although we do have a predicted protein-truncating variant, p.Arg177Ter, with nominal evidence of association to IOP and an estimated effect consistent with the missense substitutions, it is challenging to draw conclusions about its functional consequence from in silico predictions, as we have reported in earlier studies assessing when PTVs trigger degradation pathways like nonsense-mediated decay[33]. Second, it is unclear in which cell types these variants are acting on to confer the protective and IOP lowering effects. We anticipate that ANGPTL7 may be acting in the trabecular meshwork given its high expression in both adult and fetal trabecular meshwork (> 3000 FPKM)[34], we see high expression in both adult and fetal cornea (>200 FPKM), which introduces some challenges as how we interpret its functional role, and we hypothesize that given its high expression in cornea it may be one reason why we see stronger evidence of association in IOP Goldman correlated measures compared to corneal compensated IOP. Additionally, future studies should assess whether ANGPTL7 variants modify the progression of glaucoma, for example, whether ANGPTL7 carriers are less likely to go from glaucoma diagnosis to potential surgery. Although we are aggregating these data, we are thus far unable to draw definitive conclusions.*

Furthermore, we have added how this finding sits with genetic discoveries for IOP and glaucoma clarifying that this is one of the few successful rare variant associations in glaucoma and IOP. In addition, we have included some text referencing manuscripts that describe the expression levels of ANGPTL7 in trabecular meshwork cells and under steroid induction (see PMID 21199193). We have added the following text to the Discussion (page 10, lines 268-273).

> *ANGPTL7 overexpression in primary human trabecular meshwork cells was found to alter the expression of relevant trabecular meshwork proteins of the extracellular matrix, including fibronectin, collagens type I, IV, and V, myocilin, versican, and MMP1, and ANGPTL7 protein was increased as the disease progressed in POAG beagle dogs[18]. The tissue-specific protein expression data suggest that further work in dissecting the role of ANGPL7 in all possible cell types in the eye is warranted.*

What type of treatments might target the gene or its downstream effects, and how would the drug be delivered?

Given the rapidly evolving field of gene editing and siRNA, we can only speculate that if the effect is truly loss-of-function and that gene inhibition is an appropriate strategy then these therapeutic modalities may be appropriate. We have included some text in the Discussion about the type of treatments that might target the gene if it is a loss of function effect. Furthermore, delivery is a very complicated challenge. Although injection to the eye is currently commonplace in practice, it is unclear whether different therapeutic modalities, e.g. antibody, siRNA, CRISPR, base-editing would be appropriate, and whether the duration of the treatment would be sufficiently durable to be effective to prevent extremely frequent injections. New drug delivery

technologies are always of interest and clear that a durable and efficient mode of delivery would be attractive here. We clarified those points in Discussion (page 11, lines 300-316).

> *Because of the strong protective effect associated with the ANGPTL7 protein-altering variants (Supplementary Figure S9), further studies of ANGPTL7 inhibition and the specific action of these variant proteins should be useful in understanding the mechanism by which glaucoma protection occurs and whether this reveals a promising therapeutic opportunity similar to that which has been realized from the examples of PCSK9, APOC3 and cardiovascular disease[35–37]. Given the rapidly evolving field of gene editing and siRNA, we can only speculate that if the effect is truly loss-of-function and that gene inhibition is an appropriate strategy then these therapeutic modalities will be especially relevant. Therapeutic delivery is also a complicated challenge. Although injection to the eye is currently commonplace in practice, it is unclear whether different therapeutic modalities, e.g. antibody, siRNA, CRISPR, base-editing would be appropriate, and whether the duration of the treatment would be sufficiently durable to be effective to prevent extremely frequent injections or competitive against current therapeutic modalities. New drug delivery technologies are of interest and it is clear that a durable and efficient mode of delivery that mimics the protective effect of these mutations is an attractive strategy. Our genetic data from ANGPTL7 homozygotes with up to a 69% risk reduction for all glaucoma and 80% risk reduction for primary open-angle glaucoma suggest that this is likely to be a safe and effective strategy for therapeutic intervention.*

Is there a plausible explanation for the hypothesis that the authors suggest regarding modifying the glaucoma risk of patients with MYOC mutations?

It is curious that *MYOC* mutations have a strong impact on IOP and that *ANGPTL7* also has a strong effect on IOP. Unfortunately, we did not have sufficient data to assess whether *ANGPTL7* variants altered IOP in the presence of MYOC mutations (the carrier rate for both MYOC truncating allele and ANGPTL7 protein-altering variants was very rare in UK Biobank). We hope that by recalling individuals in Finland that carry both *ANGPTL7* protein-altering variant and *MYOC* truncating mutation (note the enrichment of both variants in Finland) it may help elucidate whether it also modifies IOP. Thus far, we are only able to assess it in the context of glaucoma with limited evidence (drop-in rates, but *P* of interaction is equal to 0.318). This is now included in the main text pp 8-9 lines 207-218 and in Methods pp 16 lines 488-492.

Given the Finnish enrichment of the known strong glaucoma risk allele, p.Gln368Ter, in MYOC (MAF in Finland = 0.3%, MAF in Non-Finnish European = 0.16%, reference sequence: NM_00026), we next asked whether carriers have risk reduced if they carry ANGPTL7 p.Arg220Cys. In FinnGen, we estimate that 7.0% of individuals carriers for MYOC p.Gln368Ter variant are POAG cases in comparison to 2% for non-carriers. In the presence of ANGPTL7 p.Arg220Cys, only 1.3% of individuals are POAG cases, and only 2 of 86 (2.3%) who carry both MYOC risk and ANGPTL7 protective variants were POAG cases (Supplementary Table S8). This suggests ANGPTL7 protection extends to the MYOC risk group but the small counts preclude any definitive statement regarding interaction (P = 0.318, for interaction term in a logistic regression model) - given the limited number of double-carriers, larger case-control series are needed to refine our understanding as to whether ANGPTL7 p.Arg220Cys variant modifies the glaucoma risk conferred by p.Gln368Ter in MYOC.

Interaction analysis of ANGPTL7 and MYOC

To assess whether there is an interaction between ANGPTL7 and MYOC, we performed a logistic regression analysis using R glm() function with binomial response and logit link function with an interaction term, i.e. ANGPTL7 x MYOC. We found no evidence of interaction effect, P = 0.318.

Minor comments:

- IOP is not the sole predictive factor for glaucoma

Thank you for this. We have removed the phrase “sole predictive factor” and changed it to “a predictive factor” in pp 3 lines 60-61.

Intraocular pressure (IOP) is a modifiable risk factor and predictive measure for glaucoma[1–4] (Supplementary Figure S1).

- The statement that there are "total of 68 independent loci have been unequivocally implicated in glaucoma" seems incorrect - the papers the authors cite do not reflect this on deeper reading.

We thank the reviewer. As correctly pointed out by Reviewer 1 and 2 we have added the references indicated the latest set of GWAS including Choquet et al (2018) PMID 29235454 and Hysi et al. (2014) PMID 25173106. We do note that they are IOP associations, with nominal evidence to glaucoma. Only a subset are associated with glaucoma at genome-wide significance threshold (*P* < 5x10-8). We updated the sentence (page 3, lines 63-65).

> *More than 68 independent loci have been implicated in IOP by meeting the GWAS significance threshold of association (P < 5x10-8) [5–8].*

- The text in the 2nd paragraph regarding "signals were consistently observed in left eye IOP measure" is not clear. Were previous analyses only carried on right eyes (if so, this is not clearly stated in the Results)? Are they referring to Goldmann-correlated IOP here? Results should be presented more robustly. Anyway, the authors may change their analytical approach based on the above.

We thank the reviewer. We have now updated our results to reflect both the left and right eye, and a combination of both eyes. Furthermore, we have updated our results with corneal compensated IOP as well. Those results are presented in Supplementary Table S5 in page 37. Overall, we observe through genetic correlation analysis, a high degree of correlation of genetic effects between all measurements, and also find that the proposed analysis of combining left and right eye phenotype improves power.

Reviewer #3

Reviewer #3: This is a well-written paper describes several rare ANGPTL7 protein-coding variants that are associated with lower intraocular pressure (IOP) in participants from the UK Biobank and associated with decreased risk of glaucoma in the FinnGen dataset. Several points to address:

1) The overall beta for intraocular pressure reduction by heterozygous variants is very small and even the homozygous Gln175His would not be expected be within the resolution of clinical measurement or to be clinically relevant. This should be discussed especially in regard to therapeutic development.

We would like to thank the reviewer for this comment. Although the IOP lowering effect may be limited (β = -0.53 and -0.67 mmHg for heterozygotes, -3.40 and -2.37 mmHg for homozygotes, for corneal compensated and Goldman-correlated IOP, respectively), this relatively modest IOP lowering appears to translate into a clinically meaningful 34% reduction in glaucoma risk. Further, we note that in comparison to all discovered genetic variants associated with IOP this is the strongest lowering effect we see across all GW significant associations, and is also the strongest protective effect of genome-wide significant associated variants (by comparing against all GW significant published glaucoma associations).

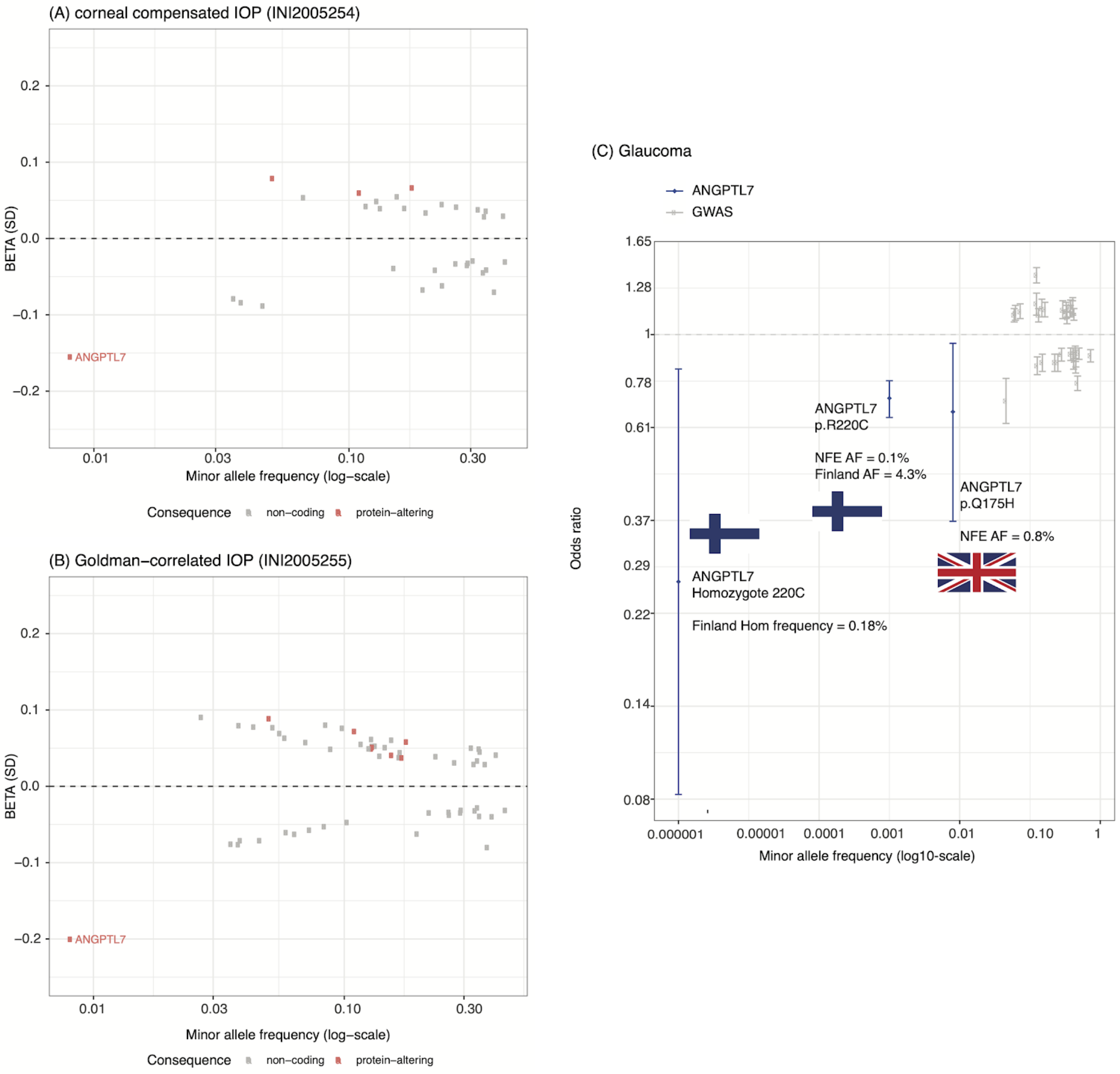

Supplementary Figure S9. The cascade plot for corneal compensated (A) and Goldman-correlated (B) intraocular pressure association analysis in UK Biobank. The cascade plot for glaucoma (C) from published genome-wide significant GWAS associations (gray) and the variants highlighted in our paper. The minor allele frequency and the BETA (SD) are plotted for the LD-pruned variants with P < 5x10-8. The odds ratios are included for LD pruned published variants with P < 5x10-8 for glaucoma.

We have included this as Supplementary Figure S9 showing that the effect size of the ANGPTL7 associated variants are stronger than previously published IOP and glaucoma associations (particularly those that lower IOP and/or lower risk for glaucoma). However, we agree that clinical relevance is a very legitimate concern and have included a detailed exploration of this point in the Discussion (Page 10 lines 280-282).

While our genetic discovery provides compelling evidence of involvement of ANGPTL7 in glaucoma, several important questions remain to be answered before its eventual clinical translation.

2) The authors note that an ANGPTL7 rare variant is likely responsible for the 1p36 signal reported in Khawaja et al. It would be interesting to note if this signal has been observed in other IOP GWAS such as Choquet et al., 2018.

We have assessed whether the IOP GWAS in Choquet et al. 2018 reports the 1p36 signal.

In Supplementary Table 8 the only reported signals in chromosome 1 are

Supplementary Table 8. Lead SNPs ($P < 5 \times 10^{-8}$) in the multiethnic meta-analysis (GERA+UKB)

					Meta-analysis (GERA+UKB)				GERA		UKB	
**Chr**	**Pos**	**SNP**	**Nearest Gene**	**Alleles**	**OR (95%CI)**	***P***	***Phet***	***I^2***	**OR (95%CI)**	***P***	**OR (95%CI)**	***P***
1	88227120	rs41461152	*LMO4/PKN2-AS1*	T/G	1.09 (1.06-1.12)	4.1×10^{-9}	0.27	17.3	1.11 (1.06-1.16)	5.7×10^{-6}	1.07 (1.04-1.11)	9.6×10^{-5}
1	103385373	rs993471	*COL11A1*	G/A	1.08 (1.05-1.11)	2.0×10^{-8}	0.98	0	1.08 (1.03-1.13)	0.00055	1.08 (1.04-1.12)	9.9×10^{-6}
1	165739598	rs2814471	*TMCO1*	C/T	1.37 (1.32-1.42)	2.0×10^{-62}	0.42	0	1.34 (1.26-1.43)	2.0×10^{-20}	1.39 (1.32-1.45)	7.5×10^{-44}

The signals are located more than 70Mb away from *ANGPTL7* (chromosome 1 1:11249399-11256039).

3) While overall the examination of the ANGPTL7 effects on MYOC368ter cases is interesting there are several questions about this result. First, since the FinnGen glaucoma cases are not actually examined, but defined by ICD codes, its possible that some of the MYOC 368ter 'noncases' are actually cases- this is particularly relevant when considering a recent study that has shown that some patients with MYOC 368ter can have glaucoma without intraocular pressure elevation (Fingert et al., JAMA Ophthalmology).

In FinnGen the ICD code for glaucoma is almost always here defined by specialty clinic - so the patients have been examined - but of course, as you say any population samples will, of course, have undiagnosed cases regardless.

Second, was the distribution of ANGPTL7 variant carriers among MYOC 368ter carriers statistically significant?

We've included analysis of the interaction of *ANGPTL7* carriers among MYOC 368ter carriers, and while it is a drop in glaucoma prevalence, we do not see evidence of significant modifying interaction (P = 0.318). This is now included in the main text pp 8-9 lines 207-218 and in Methods pp 16 lines 488-492.

Given the Finnish enrichment of the known strong glaucoma risk allele, p.Gln368Ter, in MYOC (MAF in Finland = 0.3%, MAF in Non-Finnish European = 0.16%, reference sequence: NM_00026), we next asked whether carriers have risk reduced if they carry ANGPTL7 p.Arg220Cys. In FinnGen, we estimate that 7.0% of individuals carriers for MYOC p.Gln368Ter variant are POAG cases in comparison to 2% for non-carriers. In the presence of ANGPTL7 p.Arg220Cys, only 1.3% of individuals are POAG cases, and only 2 of 86 (2.3%) who carry both MYOC risk and ANGPTL7 protective variants were POAG cases (Supplementary Table S8). This suggests ANGPTL7 protection extends to the MYOC risk group but the small counts preclude any definitive statement regarding interaction (P = 0.318, for interaction term in a logistic regression model) - given the limited number of double-carriers, larger case-control series are needed to refine our understanding as to whether ANGPTL7 p.Arg220Cys variant modifies the glaucoma risk conferred by p.Gln368Ter in MYOC.

Interaction analysis of ANGPTL7 and MYOC

To assess whether there is an interaction between ANGPTL7 and MYOC, we performed a logistic regression analysis using R glm() function with binomial response and logit link function with an interaction term, i.e. ANGPTL7 x MYOC. We found no evidence of interaction effect, P = 0.318.

4) A limitation of the study is that all the glaucoma cases are defined by ICD codes without any clinical validation. These codes used to define case-control status also include 'glaucoma secondary to eye trauma', 'secondary to eye infection or other eye disorders' and 'secondary to drugs'. Eye traumas are not genetic, while drugs causing glaucoma are primarily corticosteroids, which could drive these results considering the potential role of ANGPTL7 in steroid-responsive glaucoma (see point 6 below). Moreover, including all types of glaucoma is concerning as various forms of glaucoma have very different mechanisms and some can be difficult to distinguish without expert evaluation. Given the very high prevalence of exfoliation glaucoma in Finland this would be of special concern in the FinnGen population. Further replication of these findings in a cohort of individuals diagnosed by clinical experts would be helpful.

In FinnGen the ICD code for glaucoma is defined by specialty clinic. In FinnGen release version 4, we also have access to additional subgroup analysis on the glaucoma cases. We have also included the results from these analyses in Figure 1. Overall, we find consistent signals of protection against glaucoma subtype risk:

Analysis 2: Glaucoma analysis

6,537 cases, 170,362 controls

***ANGPTL7* p.Arg220Cys (rs147660927)**
1-11253817-C-T
MAF: 4.3% in Finland, 0.1% in UK

Glaucoma type	Cases	*P*	OR
ALL	6,537	1.9×10^{-12}	0.70
Primary open-angle	3,375	1.3×10^{-8}	0.68
Primary angle-clos.	466	0.0016	0.59
Exfoliation	1,185	6.7×10^{-5}	0.64
Normotensive	653	0.07	0.78

5) As ANGPTL7 has been shown to be increased in glaucoma secondary to steroid (glucocorticoid) exposure and this type of glaucoma has a specific ICD code is it possible to examine this subgroup among the UK Biobank cases?

Even better in patients who have been clinically diagnosed to have this type of glaucoma by glaucoma experts? Is it possible that this subgroup is driving the UKBiobank results? Showing that these variants are protective in POAG patients examined by a clinical expert with knowledge of the history of steroid exposure in the patient would also be helpful.

The steroid-induced glaucoma is rare compared with POAG (or exfoliation glaucoma), thus it is fair to reason that the signal comes from the patients with POAG. (In FinnGen, we cannot examine just steroid-induced cases, because we do not have ICD-10 code for specifically for that. The H40.6 drug-induced glaucoma or H40.5 glaucoma due to other eye disease could work as a surrogate, but it is not specifically steroid-induced).

6) There is very little discussion of any potential protective role for the ANGPTL7 protein or functionally how loss of function variants could impact intraocular pressure and glaucoma. Additionally, while the nonsense variant is likely to be loss of function, this may not actually be the case as the most common MYOC variant (368ter) is actually a gain of function. Its not clear if the missense alleles are loss or gain of function. Again, similar to MYOC the missense alleles

are gain of function. This information is very relevant to the development of ANGPTL7 based therapies.

We thank the reviewer for this comment. As reviewer #1 and #2 suggested we have added additional discussion text on the potential protective role for the ANGPTL7 protein and functionally how loss of function variants could impact intraocular pressure and glaucoma. We also see it as the next step to mechanistically dissect how these variants are introducing the protective effect on glaucoma and lowering effect on IOP levels. Those points are now clarified in Discussion (pages 10-11, lines 280-295).

> *While our genetic discovery provides compelling evidence of involvement of ANGPTL7 in glaucoma, several important questions remain to be answered before its eventual clinical translation. First, we were not able to assess whether the missense variants are complete loss-of-function, partial loss-of-function variants, dominant negative, or gain of function given the data we have at hand. Although we do have a predicted protein-truncating variant, p.Arg177Ter, with nominal evidence of association to IOP and an estimated effect consistent with the missense substitutions, it is challenging to draw conclusions about its functional consequence from in silico predictions, as we have reported in earlier studies assessing when PTVs trigger degradation pathways like nonsense-mediated decay[33]. Second, it is unclear in which cell types these variants are acting on to confer the protective and IOP lowering effects. We anticipate that ANGPTL7 may be acting in the trabecular meshwork given its high expression in both adult and fetal trabecular meshwork (> 3000 FPKM)[34], we see high expression in both adult and fetal cornea (>200 FPKM), which introduces some challenges as how we interpret its functional role, and we hypothesize that given its high expression in cornea it may be one reason why we see stronger evidence of association in IOP Goldman correlated measures compared to corneal compensated IOP.*

7) This sentence is confusing, "Given these findings, we next asked whether any of these putative IOP-lowering genetic variants showed effects consistent with reducing glaucoma risk in an independent set of unrelated British individuals that do not have IOP measures (4,269 cases and 251,355 controls)." Are these glaucoma cases not included in the set of UK Biobank individuals with eye phenotype data?

We have clarified the statement to specify that these are glaucoma cases not included in the set of UK Biobank individuals with eye phenotype data. These are indeed two disjoint sets of individuals: 1) One for IOP genetic discovery, and 2) for glaucoma (where IOP measurements are not available) to ensure that we were not introducing any bias by including overlapping samples. We have updated the main text (pages 7-8, lines 171-181).

We next asked whether any of these putative IOP-lowering genetic variants showed effects consistent with reducing glaucoma risk. We focused on unrelated White British individuals that do not have IOP measures (4,238 cases and 250,660 controls, Supplementary Figure S6). For p.Gln175His in ANGPTL7, using logistic regression analysis with age, sex, and principal components (PC1-PC4) as covariates, we estimated that the variant lowers glaucoma risk by 34% (P = 0.00543; OR = 0.66 [95% CI: 0.366 - 0.954], Table 2). The three additional protein-altering variants did not significantly confer protection against glaucoma (burden test P = 0.77). This is consistent with power calculations, using Genetic Power Calculator[15], where our power to detect association for rare variants with a composite allele frequency of 0.345% and a binary trait in 4,238 cases and 250,660 controls at alpha = 0.05, and 0.001, i.e. P < .05 and .001, with OR of 0.7 (30% reduction in risk) is equal to 62% and 15.2%, respectively.

Furthermore, we have added a new figure, Figure 1, describing the overview of the study and clarified that the two sets of individuals are disjoint.

Analysis 1: Rare protein-altering variants genome-wide association scan

Intraocular pressure analysis (n = 82,253 individuals)
41,590 genotyped variants; 14,368 genes

$P < 10^{-6}$

***ANGPTL7* p.Gln175His (rs28991009)**
1-11253684-G-T
P = corneal compensated | Goldman-correlated
Combined Left and Right eye P = 5.96×10^{-9} | 1.07×10^{-13}

$P < 2.5 \times 10^{-6}$

***ANGPTL7* (burden test, dispersion test) ALL; without p.Gln175His**
P = corneal compensated | Goldman-correlated
Combined Left and Right eye P = (1.88×10^{-7}, 1.43×10^{-8}); (0.11, 0.24) | (1.44×10^{-14}, 2.89×10^{-15}); (3.70×10^{-4}, 1.20×10^{-3})

Test hypothesis: *ANGPTL7* in Glaucoma

Analysis 2: Glaucoma analysis

6,537 cases, 170,362 controls

***ANGPTL7* p.Arg220Cys (rs147660927)**
1-11253817-C-T
MAF: 4.3% in Finland, 0.1% in UK

Glaucoma type	Cases	*P*	OR
**ALL**	6,537	1.9×10^{-12}	0.70
**Primary open-angle**	3,375	1.3×10^{-8}	0.68
**Primary angle-clos.**	466	0.0016	0.59
**Exfoliation**	1,185	6.7×10^{-5}	0.64
**Normotensive**	653	0.07	0.78

Analysis 3: Glaucoma analysis in individuals without IOP measurements (not in Analysis 1)

4,238 cases, 250,660 controls

p.Gln175His (rs28991009), 1-11253684-G-T
$P = 6.2 \times 10^{-3}$, OR = 0.67

***ANGPTL7* (burden test, dispersion test)**
ALL; without p. Gln175His
P = (0.0129, 8.7×10^{-3}) ; (0.72, 0.72)
